# Cross-feeding modulates the rate and mechanism of antibiotic resistance evolution in a model microbial community of *Escherichia coli* and *Salmonella enterica*

**DOI:** 10.1371/journal.ppat.1008700

**Published:** 2020-07-20

**Authors:** Elizabeth M. Adamowicz, Michaela Muza, Jeremy M. Chacón, William R. Harcombe

**Affiliations:** 1 Department of Genetics, Cell Biology, and Development, University of Minnesota, Minneapolis, Minnesota, United States of America; 2 Department of Ecology, Evolution and Behavior, University of Minnesota, St. Paul, Minnesota, United States of America; 3 BioTechnology Institute, University of Minnesota, St. Paul, Minnesota, United States of America; National Institutes of Health, UNITED STATES

## Abstract

With antibiotic resistance rates on the rise, it is critical to understand how microbial species interactions influence the evolution of resistance. In obligate mutualisms, the survival of any one species (regardless of its intrinsic resistance) is contingent on the resistance of its cross-feeding partners. This sets the community antibiotic sensitivity at that of the ‘weakest link’ species. In this study, we tested the hypothesis that weakest link dynamics in an obligate cross-feeding relationship would limit the extent and mechanisms of antibiotic resistance evolution. We experimentally evolved an obligate co-culture and monoculture controls along gradients of two different antibiotics. We measured the rate at which each treatment increased antibiotic resistance, and sequenced terminal populations to question whether mutations differed between mono- and co-cultures. In both rifampicin and ampicillin treatments, we observed that resistance evolved more slowly in obligate co-cultures of *E*. *coli* and *S*. *enterica* than in monocultures. While we observed similar mechanisms of resistance arising under rifampicin selection, under ampicillin selection different resistance mechanisms arose in co-cultures and monocultures. In particular, mutations in an essential cell division protein, *ftsI*, arose in *S*. *enterica* only in co-culture. A simple mathematical model demonstrated that reliance on a partner is sufficient to slow the rate of adaptation, and can change the distribution of adaptive mutations that are acquired. Our results demonstrate that cooperative metabolic interactions can be an important modulator of resistance evolution in microbial communities.

## Author summary

Little is known about how ecological interactions between bacteria influence the evolution of antibiotic resistance. We tested the impact of metabolic interactions on resistance evolution in an engineered two-species bacterial community. Through experimental and modeling work, we found that obligate metabolic interdependency slows the rate of resistance acquisition, and can change the type and magnitude of resistance mutations that evolve. This work suggests that resistance evolution may be slowed by targeting both a pathogen and its metabolic partners with antibiotics. Additionally, we showed that community context can generate novel trajectories through which antibiotic resistance evolves.

## Introduction

The ability of pathogens to rapidly evolve antibiotic resistance is a pressing global challenge. While resistance frequently evolves in complex microbial communities, relatively little is known about how species interactions influence the evolution of antibiotic resistance [1–3]. Most of the studies that do incorporate multiple species focus more on the role of horizontal transfer of antibiotic resistance between species, and less on the *de novo* evolution of resistance within genomes [3–7]. Additionally, antibiotic resistance studies in multi-species systems typically involve unknown interactions between species, with some exceptions in modelling [8–11]. The role of specific interspecies interactions in the evolution of antibiotic resistance in microbial communities, therefore, remains largely unexplored.

Positive interactions are common in bacterial communities [12]. One important type of positive interaction is cross-feeding, wherein two species exchange essential metabolites [13]. The resilience of metabolically interdependent microbial systems to environmental disturbances is a growing field of study, with research being conducted into how these systems resist invasion [14,15] and respond to abiotic environmental changes [8,16–18]. We have previously shown that obligate cross-feeding influences the effect of antibiotics over short time scales (i.e. within a single growth curve): the least resistant member of an obligate cross-feeding community constrains the ability of more resistant community members to grow at high antibiotic concentrations, producing “weakest-link” dynamics [18]. In this case, antibiotic resistance in monoculture (genetic mechanisms conferring an ability to grow at higher antibiotic concentrations) did not predict the sensitivity in co-culture (a phenotypic trait describing ability to grow at high antibiotic concentrations), as the sensitivity was limited by the dependence on the least resistant species. This idea has also been demonstrated by others through modelling approaches [8].

We hypothesize that the weakest link pattern described above should also hold over evolutionary timescales; that is, at any given point during the evolution of resistance in a metabolically interdependent community, one ‘weakest-link’ species should set the antibiotic sensitivity of the whole community. The obligate cross-feeding interactions would then require that the weakest link species evolve resistance in order for the whole community to rise in the concentration of antibiotic at which growth is possible. We therefore hypothesize that metabolically interdependent communities will be slower to adapt to rising antibiotic levels than their single-species counterparts. Importantly, in this study, we differentiate between sensitivity (the ability of a species or community to grow at a certain antibiotic concentration) and resistance (a genetic change or mechanism that modulates tolerance) [19]. This distinction is important as we have previously shown that obligate mutualistic interactions can decouple sensitivity and resistance when a highly resistant species is dependent on a less resistant one [20].

We also hypothesize that weakest link dynamics should affect the mechanisms of resistance evolution. Cross-feeding should change the benefit of some mechanisms; for example, the evolution of shared resistance through antibiotic-degrading enzymes [3,21,22] or induction of resistance in a partner species [23,24] could be uniquely selected for in co-culture. Cross-feeding may also make some resistance mechanisms more costly. For instance, altering cell wall permeability may be particularly maladaptive in cross-feeding communities, where exchanged nutrients will typically be at low concentrations [25–27]. Finally, a reduction in the rate of adaptation may drive different mechanisms of resistance to evolve. Varying the rate at which antibiotics are increased has been shown to lead to different evolutionary trajectories [28]. More rapid changes in antibiotic concentration tend to select for mutations with larger effects that are more costly [28]. These big effect mutations can trap populations on sub-optimal fitness peaks [28]. We therefore hypothesize that we will see different mechanisms of resistance evolve in co-culture vs. monoculture, though the exact nature of these differences is unclear.

We investigated whether obligate cross-feeding altered the rate and mechanism of antibiotic resistance evolution. We used a previously engineered two-species system wherein a methionine-auxotrophic *E*. *coli* consumes lactose and excretes carbon by-products that *S*. *enterica* uses as a carbon and energy source, and *S*. *enterica* overproduces the methionine required by *E*. *coli*. We evolved six replicate populations of each species growing in monoculture (providing *E*. *coli* with lactose and methionine, and *S*. *enterica* with glucose) and in obligately cross-feeding co-culture (providing both species with lactose only, hereafter referred to as ‘co-culture’) along identical antibiotic gradients of rifampicin or ampicillin. At each transfer, the MIC (minimum inhibitory concentration) was assessed. We also constructed a mathematical model of resistance and used it to assess the generality of our findings, specifically with respect to changes in population size or physiology between monocultures and co-cultures. Our results show that growth in an obligate co-culture slows the rate of adaptation, and sometimes leads to different mechanisms of resistance.

## Results

### Antibiotic resistance evolves more quickly in monoculture than in obligate co-culture

**Fig 1:**
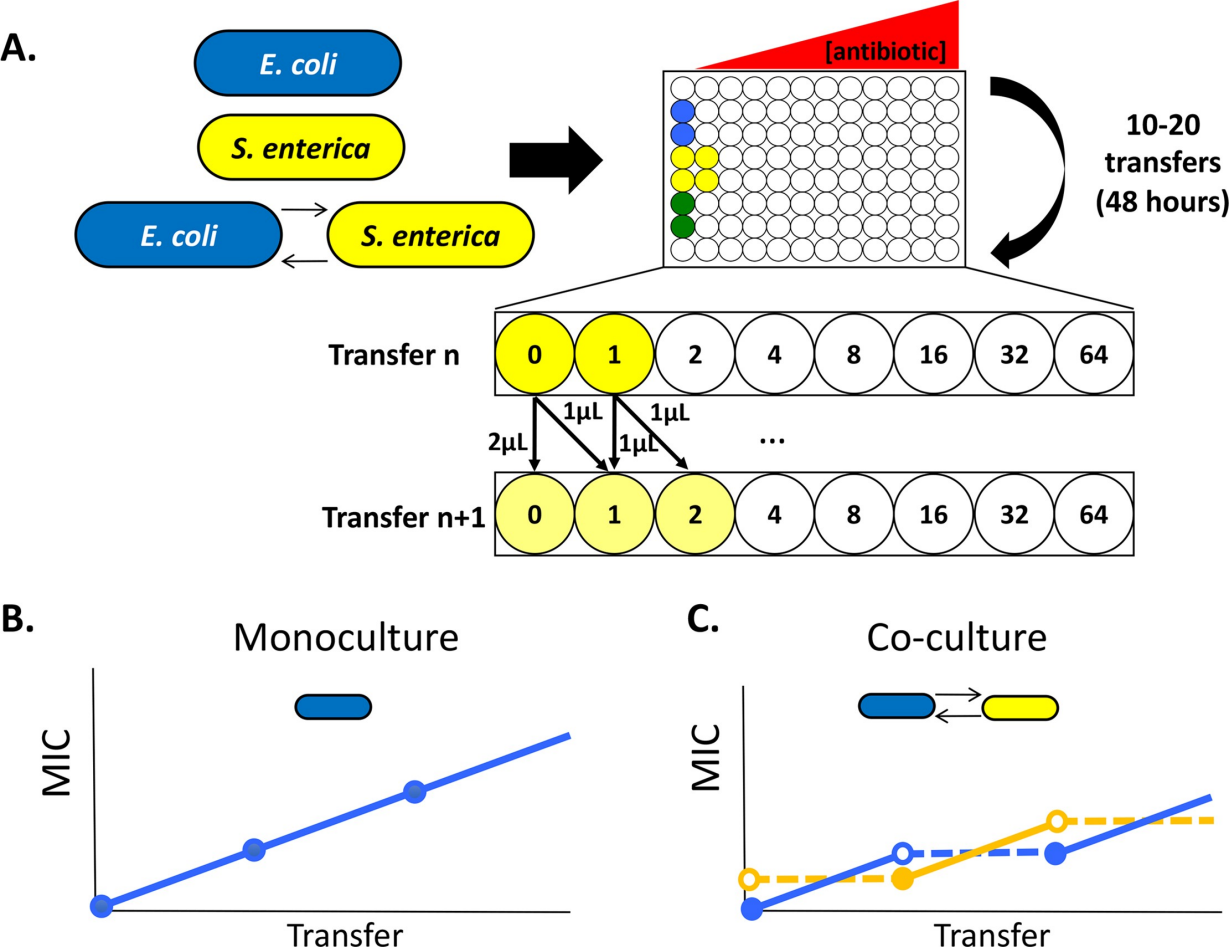
Schematic of experimental setup and expectations. **A.** Monocultures of *E*. *coli* (blue) and *S*. *enterica* (yellow), as well as cross-feeding co-cultures (green), were distributed in six replicate populations along an antibiotic gradient in 96-well plates. The antibiotics tested included rifampicin and ampicillin, and the concentration of antibiotic increased twofold at each well. 96-well plates were incubated with shaking at 30°C for 48 hours, then cells were transferred to fresh medium and antibiotic in a new plate. The transfer regime was to transfer 1μL of culture to a fresh well containing the same concentration of antibiotic at which it had previously grown, and 1μL to a fresh well containing one concentration step higher antibiotic. At each passage, the OD600 of the plate was measured, as well as species-specific fluorescence (CFP for *E*. *coli*, YFP for *S*. *enterica*). **B-C.** Hypothesis on why time under selection may be sufficient to explain MIC differences between monocultures and co-cultures. **B.** In monoculture, each species is under selection at every time step, thus selecting for increasing resistance with each passage. **C**. In obligate co-culture, only the more antibiotic-sensitive species is under selection at a given time, and effective co-culture resistance requires an increase in MIC in both species. This leads to the slower rise in resistance in the co-culture.

First, we tested the rate at which antibiotic resistance evolved in monocultures of *E*. *coli* and *S*. *enterica* as well as obligate co-cultures of the two species. We established six replicate cultures of each monoculture, and the co-culture. Each culture was distributed along an antibiotic gradient of either ampicillin or rifampicin, with cells initially inoculated into each antibiotic concentration along the gradient. After 48 hours of growth, we transferred cells from all wells in the gradient in a 1/200 dilution to fresh medium in the same antibiotic concentration, as well as double that concentration (**Fig 1A**). The same antibiotic gradient and transfer regime was used for both monocultures and co-cultures. Replicates may ultimately end up experiencing different maximum antibiotic concentrations, but this is because of differences in evolved resistance allowing them to move faster up the gradient, rather than externally imposed differences in gradients. We transferred populations for 20 transfers (approximately 150 generations). At each transfer, we measured total population density (by OD600) and calculated MIC_90_ based on the density in wells along each gradient. After the final transfer, three colonies of each species were isolated from each replicate population and their MIC_90_ values measured to assess the correlation between specific mutations and growth rate, yield, and MIC. Due to contamination, we removed one replicate of rifampicin-evolved *S*. *enterica* in monoculture and conservatively only used data out to transfer 10 (75 generations) for the ampicillin-evolved lines. There was no significant difference in *E*. *coli* population size in monoculture and co-culture (p = 0.43), while in *S*. *enterica* the co-culture was 42% smaller than the monoculture (p < 0.001, **S1 Fig**).

**Fig 2:**
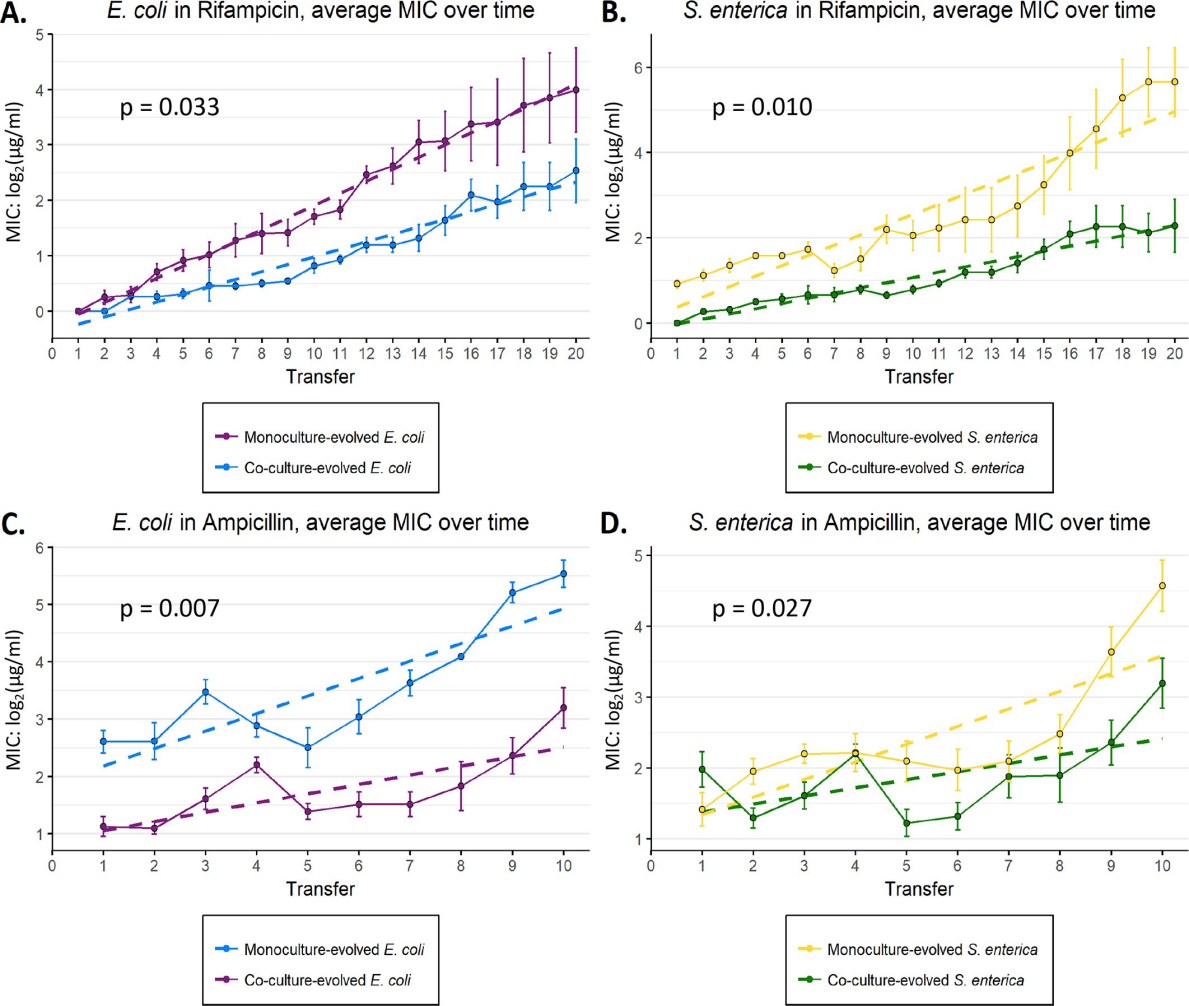
Resistance evolves more slowly in co-culture-evolved populations vs. monoculture-evolved populations. Six replicate populations each of monocultures and co-cultures were evolved along a rifampicin gradient (**A-B**) or an ampicillin gradient (**C-D**); gradients were identical for monocultures and co-cultures. Population MICs for each species (*E*. *coli* **A, C**; *S*. *enterica* **B, D**) were measured each transfer and the resulting MICs plotted. Statistical analysis was performed using a mixed-effects model with a randomized slope for each replicate within a culture type. The fitted slopes for each treatment are indicated by the dashed lines. P-values are for the interaction term between passage and culture type. Error bars represent the standard deviation of MIC among the six replicates.

We found that resistance evolved more quickly in monoculture populations than in co-cultures (**Fig 2A and 2B**). We used a mixed effects model to analyze the rate at which MIC doubled in each replicate (i.e. the response variable was log_2_(MIC)). The model included a fixed effect of treatment, a fixed effect of time, and a fixed effect of the interaction between treatment and time (it also included a random effect of replicate to control for structure resulting from repeated measures, see Methods). If monocultures and co-cultures evolved resistance along the same trajectory, but only began at different points along that trajectory, then we would expect to observe a significant main effect of treatment on log_2_(MIC), but not a significant interaction between treatment and time. A significant increase in the doubling rate unaffected by treatment would appear as a significant main effect of time but no interaction. Finally, a difference in the doubling rate of MIC by treatment would be apparent as a significant treatment:time interaction, regardless of either significant main effect. In rifampicin, growth in each monoculture was associated with a significantly higher increase in MIC per transfer than growth in co-culture (treatment:time interaction term p = 0.0328 for *E*. *coli*, treatment:time interaction term p = 0.0100 for *S*. *enterica*). In ampicillin, resistance in both species also rose more quickly in monoculture than co-culture, even over just ten transfers. The per-transfer increase in MIC of both monocultures was higher than that of co-cultures (treatment:time interaction term p = 0.007 for *E*. *coli*, treatment:time interaction term p = 0.0272 for *S*. *enterica*) (**Fig 2B**).

### Similar mechanisms of rifampicin resistance evolve in monoculture and obligate co-culture

To determine whether cross-feeding selected for different resistance mechanisms we sequenced resistant populations at the end of the experiments. We extracted genomic DNA from the well that grew at the highest concentration of antibiotic for each replicate population. For each resistant population we created a list of mutations and their frequencies, excluding any mutations also observed in antibiotic free controls (**S1 Table and S2 Table**). For further analysis, we focused on genes that acquired mutations in more than one replicate within a treatment, as parallel evolution is a signature of adaptation [29].

**Fig 3:**
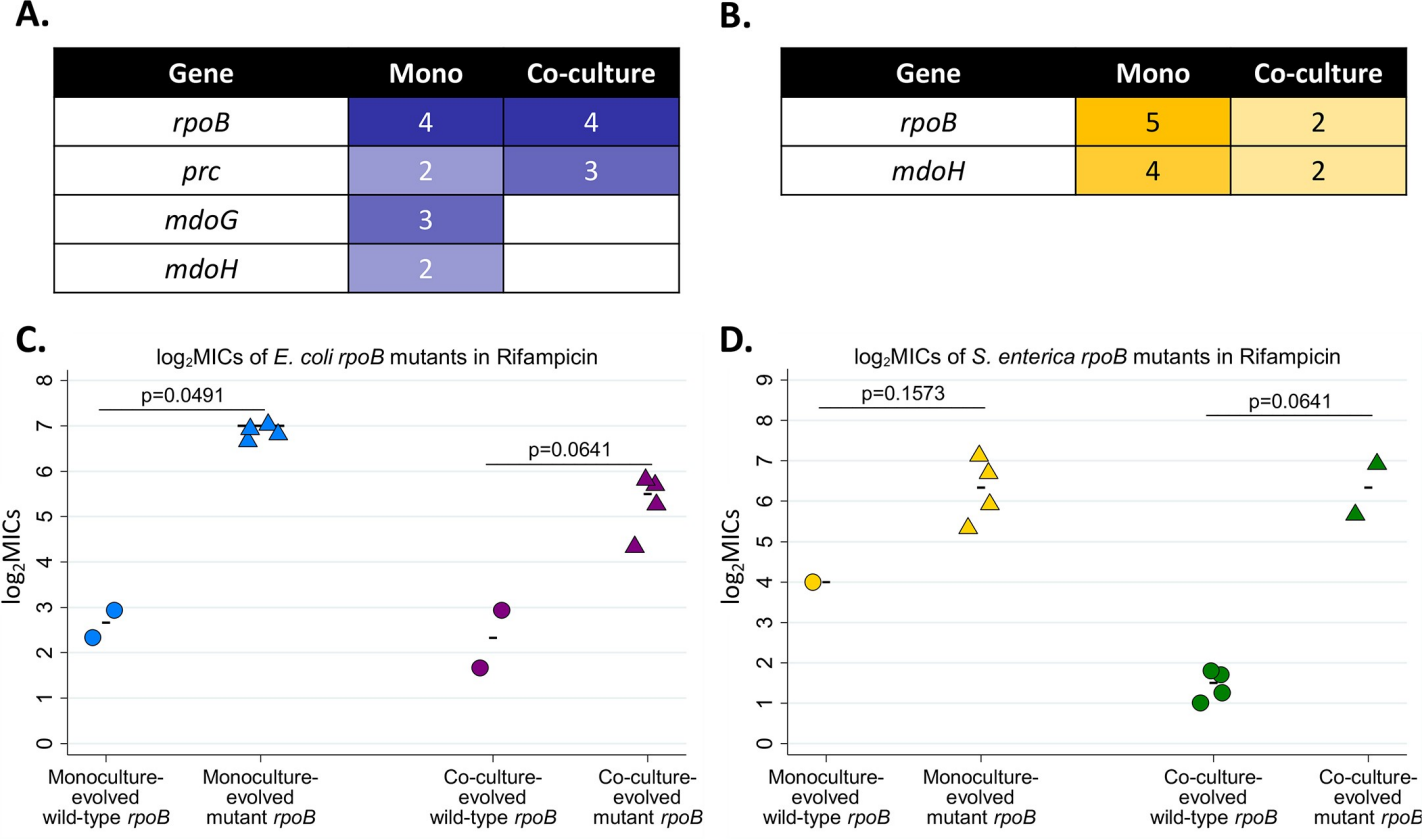
Resistance-associated mutations in rifampicin-resistant evolved populations **A-B.** Lists of mutations which arose in *E*. *coli* (**A**) or *S*. *enterica* (**B**) monocultures and co-cultures. The number in each box represents the number of independent replicates in which the putative resistance mutation was observed. Additional mutations that occurred in only one population, and details on the nature of these mutations, may be found in **S1 Table**. **C-D.** Rifampicin MICs for isolates with wild-type vs. mutant *rpoB* genes in *E*. *coli* (**C**) and *S*. *enterica* (**D**). Isolates were obtained from passage 20 populations by streaking onto selective medium and picking isolated colonies. Each data point represents the average MIC for three isolates obtained from a single population. For each species- culture type combination, there are six populations total, and the statistical comparisons represent MIC comparisons between populations with wild type vs. mutant alleles. Note that there are only five *S*. *enterica* monoculture populations due to possible contamination in one population. MIC_90_ values for isolates were defined as the lowest concentration of antibiotic which decreased growth by greater than 90% by 48 hours at 30°C. Additionally, monoculture lines evolved more mutations than co-culture lines. Mutations in *mdoG* and *mdoH* were observed in both monocultures of *E*. *coli* and *S*. *enterica* under rifampicin selection, but at much lower frequencies in co-culture (**S1 Table, S2 Fig**). All but one of these mutations were insertions or deletions of 1-6bp causing frameshift mutations (**S1 Table).** Both *mdoG* and *mdoH* likely influence cell membrane permeability [31,32]. The mutations were not associated with any changes in monoculture or co-culture growth rates (**S3 Fig**). Taken together, the pattern of rifampicin resistance mutations suggests that populations were moving along the same evolutionary trajectory in monoculture and co-culture, but progressed further along that trajectory in monocultures.

Under rifampicin pressure, the genes that acquired mutations in co-culture were a subset of those that acquired mutations in monoculture for both species (**Fig 3A and 3B**). The most clearly identifiable resistance-associated mutation was *rpoB*, a component of RNA polymerase and the most common mutational target for rifampicin resistance [30]. Mutations in this gene arose in both species, in four out of the six replicates each in monoculture and co-culture (**Fig 3A**) and were strongly tied to higher levels of resistance (**Fig 3C and 3D**). All *rpoB* mutations were nonsynonymous single base pair substitutions. Interestingly, *E*. *coli* monoculture lines all evolved different *rpoB* mutations, but all coculture *E*. *coli* evolved the same S574Y mutation **(S1 Table)**. A mutation in a prophage tail-specific protein, *prc*, of *E*. *coli* was also repeatedly observed in both monoculture and co-culture (**S2 Fig);** all mutations were short (2-11bp) deletions **(S1 Table)**. The overlap in mutations suggests that co-cultures and monocultures evolved rifampicin resistance along similar adaptive trajectories, albeit at different rates.

### Different mechanisms of ampicillin resistance arise in monocultures vs. obligate co-cultures

In contrast to our results from rifampicin, the mutational spectra that we obtained from sequencing ampicillin-resistant populations suggests different resistance mechanisms arose in monoculture vs. co-culture. We saw a greater variety of genes acquiring adaptive mutations across replicates in ampicillin relative to rifampicin treatments. Nevertheless, we observed distinct mutational signatures in monocultures and co-cultures.

**Fig 4:**
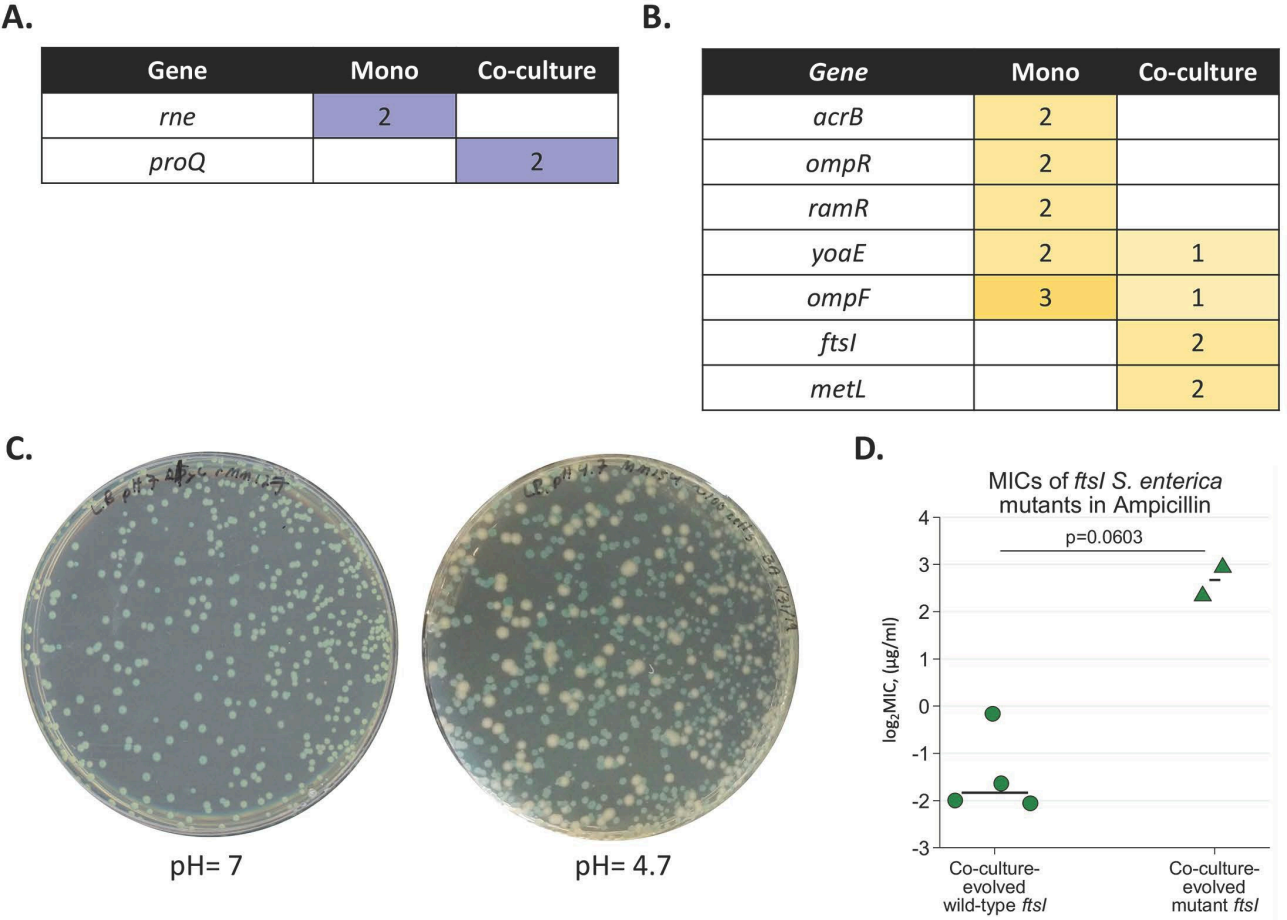
Resistance- associated mutations in ampicillin-resistant evolved populations **A-B.** Lists of mutations which arose in *E*. *coli* (**A**) or *S*. *enterica* (**B**) monocultures and co-cultures. The number in each box represents the number of independent replicates in which the putative resistance mutation was observed. Additional mutations that occurred in only one population, and details on the nature of these mutations, may be found in **S1 Table**. **C.** Image of co-culture populations containing *ftsI* mutations on Petri plates with LB pH = 7 (left) and pH = 4.7 (right). Blue colonies are *E*. *coli* which metabolize X-gal to a blue color; white colonies are *S*. *enterica*. No white colonies were observed on LB at pH = 7. **D.** MICs of isolates from libraries containing *ftsI* mutations in pH = 4.7 medium. Isolates were obtained from passage 10 populations by streaking onto selective medium and picking isolated colonies. Each data point represents the average MIC of three isolates from a single population. For each species- culture type combination, there are six populations total, and the statistical comparisons represent MIC comparisons between populations with wild type vs. mutant alleles MIC_90_ values for isolates were defined as the lowest concentration of antibiotic which decreased growth by greater than 90% by 48 hours at 30°C. Each point represents the average MIC of three isolates from a single population. P = 0.0603, Mann-Whitney U.

Very few genes acquired mutations more than once in the *E*. *coli* replicates. Two co-culture replicates evolved unique mutations in *proQ*, a regulator of efflux pumps [33] **(Fig 4A, S1 Table**). In monoculture, a gene associated with stress response (*rne*) contained an identical mutation in two populations (**S1 Table**). Given the relative paucity of mutations there is at best only a weak signature of differential resistance mechanisms evolving in *E*. *coli* in monoculture and co-culture.

Compared to *E*. *coli*, there was a larger number of *S*. *enterica* genes that acquired mutations only in monoculture or in coculture, but not both. (**Fig 4B, S1 Table**). Mutations in efflux pump genes (*acrB*, *ramR*) and cell permeability (*ompR*) arose in *S*. *enterica* monoculture but not in co-culture. The *ompF* mutations in monoculture were caused by an IS10 insertion and the *yoaE* mutations by an intergenic inversion (**S2 Table**). None of these mutations were associated with a significant increase in resistance except for *ramR*, wherein mutants had a higher median MIC than wild-type isolates (p = 0.0491, **S4 Fig**). Conversely, mutations in *ftsI*, a penicillin-binding protein (PBP3), were only observed in co-culture. *ftsI* is thought to be an essential gene and is known to be a target site for β-lactam antibiotics [34]. Interestingly, the mutations we observed in *ftsI* in our whole-genome sequencing were a combination of point mutations (D534Y at 75% frequency in rMM158) and mutations which should have ablated gene function (+A at 100% frequency in rMM127 and Q142* in rMM158) (**S1 Table**). We found that we could not isolate *S*. *enterica* clones with these mutations from evolved populations spread on typical LB agar, except for rare isolates that also had a suppressor mutation in the same codon as the Q142* mutation site which eliminated the stop codon (**S1 Table).**

As *ftsI* is an essential gene [35], it was perhaps not surprising that isolating loss-of-function mutations was difficult. However, this then raised the question of how *ftsI* loss-of-function mutants could rise to high frequency in the co-cultures. Castanheira et al. recently demonstrated that the lethality associated with *ftsI* loss in *S*. *enterica* could be mitigated by growing *S*. *enterica* under acidic conditions (pH<5.8); under these conditions, a second PBP3, which they called PBP3_SAL_, is expressed [35]. In light of this, we plated co-culture populations containing Q142* or +A mutations in *ftsI* on LB of pH 4.7. On acidic plates we were able to obtain *S*. *enterica* isolates at roughly equal frequencies to what we would expect in the population, and Sanger sequencing demonstrated that these isolates did contain the loss-of-function Q142* or +A mutations and no secondary repressor mutations. These isolates did not show detectable growth in isolation unless the growth medium was acidified. They were associated with increased MICs versus isolates with wild-type *ftsI*, though this difference was not statistically significant due to low power (**Fig 4D**, p = 0.0603). Perplexingly, the co-culture media in which the *ftsI* mutants evolved did not show detectible pH changes, suggesting some novel induction mechanism for PBP3_SAL_ in our co-cultures. Interestingly, *ftsI* mutant isolates had co-culture growth rates comparable to wild-type when paired with an *E*. *coli* ancestor (**S5 Fig**). This suggests that *ftsI* knockout mutations were non-viable in monoculture but conferred little cost in co-culture.

### A model suggests that interdependency is sufficient to generate differences in the rate of evolution of resistance

To test the effect of interdependence on evolution of antibiotic resistance in the absence of species-specific biological details, we developed a simple model. The model is based upon the haploid Wright-Fisher model with selection. Each species was kept at a density of 1000 individuals per well (regardless of how many species were present in a well) and transferred every ten generations. Genotypes within a species had two alleles: one conferring a growth rate, which affected their reproductive success between generations, and one conferring antibiotic resistance, which determined whether they could grow at all in the well. Each generation mutations could occur which could alter either growth rate or MIC. The transfer regime mimicked our experiments and allowed populations to progress out along an antibiotic gradient as they evolved resistance. At the beginning of growth after a transfer, at least one genotype from each inter-dependent species must be resistant to the present antibiotic concentration for any growth to occur. This interdependency allowed us to model cross-feeding, wherein one species cannot grow without metabolites supplied by the other, but is also representative of other types of mutualism. Importantly, this model allowed us to control for population size and physiology between monocultures and co-cultures, which we were unable to do in our experimental setup. This model therefore allowed us to test whether interspecies interdependence was sufficient to drive differences in rate of resistance evolution.

**Fig 5:**
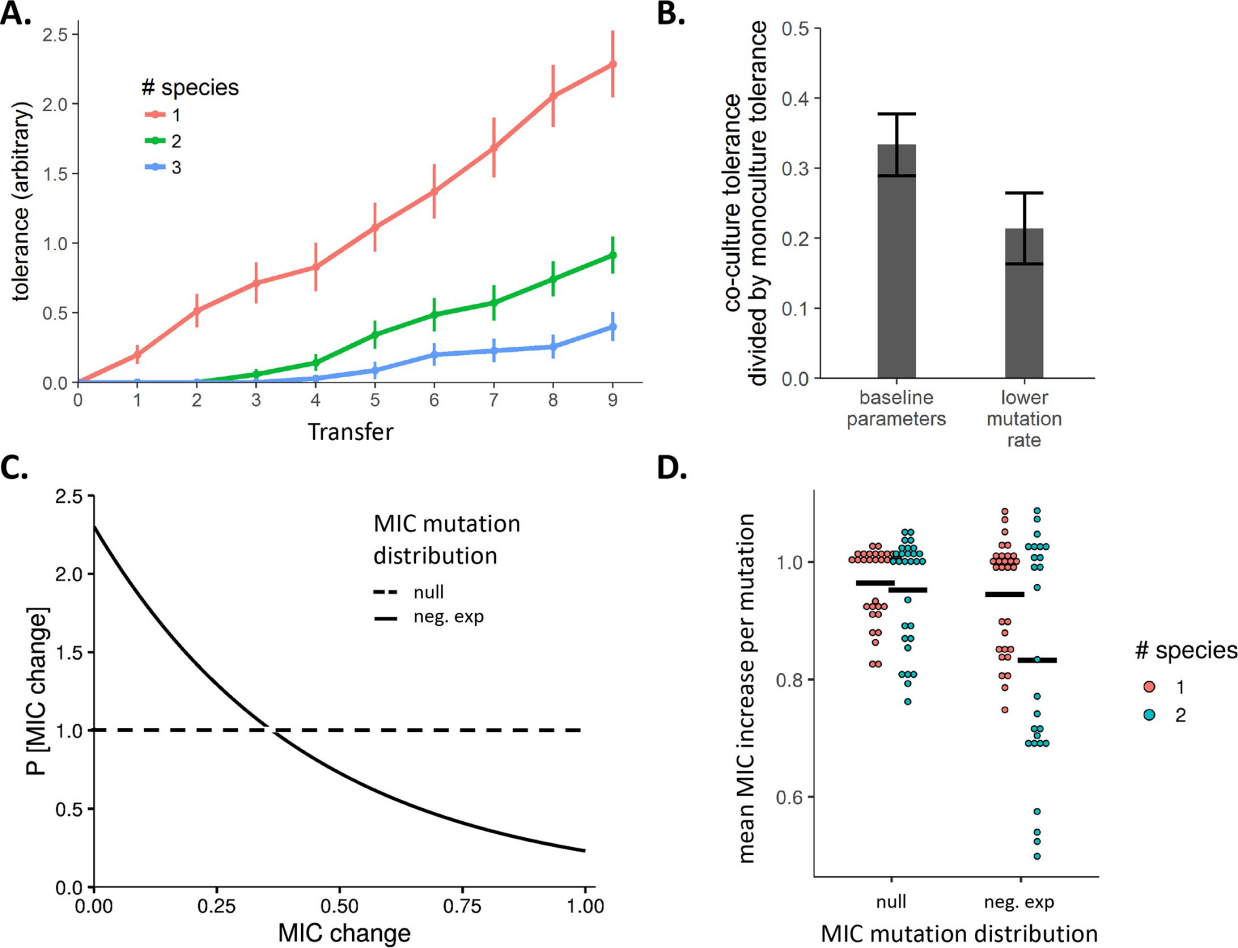
Simulation model of the evolution of antibiotic resistance in single-species vs. multi-species obligately dependent communities. **A.** A simulated evolution experiment with 35 replicate populations shows that increasing the number of interdependent species in a consortium results in a slower increase in the average MIC over time. Error bars represent the standard deviation among replicates. **B.** Effect of lower mutation rate on resistance evolution in model. Lower mutation rates result in bigger differences in the relative tolerance of one-species versus two species systems. **C.** Distributions from which MIC-altering mutations were randomly pulled. The x-axis is relative to the maximum MIC change possible in one mutation. The “null” model used a uniform distribution. The “neg exp” used a truncated negative exponential distribution with rate parameter 2.3, which kept 90% of random numbers less than the max MIC change of 1.0. **D.** Mean MIC increase conferred per mutation as a function of the mutation distribution and the number of species.

We first examined the relationship between the number of interdependent species and the rate of evolution of antibiotic resistance. We found that antibiotic tolerance (the highest antibiotic concentration at which a species grew) evolved more slowly as more interdependent species were simulated (**Fig 5A,** linear mixed-effects model, t = -9.4, p < 1e-7). When the mutation rate was reduced in all populations, cross-feeding more strongly suppressed the rate at which resistance evolved (**Fig 5B,** one-way ANOVA, F(2,297) = 6.9, p = 0.0012). Cross-feeding also reduced the number of mutations that accumulated within a population (**S6 Fig**, F(2,102) = 20.98, p < 1e-7).

We next tested how the distribution of mutations influenced the mechanisms of resistance. We simulated evolution when all mutations were equally likely versus when mutations which conferred a greater resistance were less common, using a negative exponential distribution **(Fig 5C)**. Under a uniform distribution there was no difference in the average size of resistance mutations accumulated in monocultures and co-cultures **(Fig 5D**). However, when big-effect mutations were rare, populations in co-cultures acquired significantly smaller effect mutations than populations in monoculture (**Fig 5D**, two-way ANOVA, interaction between # of species and mutant distribution: F(1,111) = 5.087, p = 0.026; Tukey's HSD finds only neg. exp with 2 species is different from other treatments). Our results demonstrate that when the frequency of mutations is not uniform, populations in monoculture and co-culture will sample different sets of mutations.

## Discussion

Our results demonstrate that obligate cross-feeding can slow the rate of antibiotic resistance evolution, and in some contexts change the mechanisms through which resistance evolves. Relative to monoculture, obligate cross-feeding slowed the rate of adaptation of both *E*. *coli* and *S*. *enterica* in the face of two different drugs. In rifampicin, genes that acquired resistance mutations in co-culture were a subset of the genes involved in adaptation in monoculture. In ampicillin, in contrast, we observed different resistance mechanisms arising in monoculture and in co-culture. Additionally, we used a model to demonstrate that mutualism may generally slow the rate at which populations evolve resistance. Critically, the model demonstrates that interdependency is sufficient to alter rates and mechanisms of evolution, independent of differences in population size or physiology. Overall, our experimental and modeling results showed that monocultures evolved more resistance than co-cultures, irrespective of species and antibiotic identity, suggesting that slower resistance evolution in cross-feeding co-cultures is a general phenomenon. These results demonstrate the importance of taking ecological context into account when studying the evolution of antibiotic resistance, and our work adds to a growing body of literature on how species interactions shape evolutionary rates in microbial communities [36,37].

We have shown that mutualistic interactions between species can result in slower evolution of antibiotic resistance. This reduced rate of adaptation to antibiotics was highly robust appearing for both bacteria under both drug treatments. However, it is important to note that populations in co-culture experienced additional differences which could have influenced rates of adaptation. For example, in co-culture *S*. *enterica* likely consumes a mix of carbon sources while in monoculture it was provided only glucose. Carbon source can alter the evolution of resistance [38]. *S*. *enterica* also had a smaller population in monoculture and previous work has suggested that changes in population size are an important mechanism through which species interactions may influence evolutionary rate [36,37]. However, we do not think that the differences in rate were driven solely by these effects in our experiments for two reasons. First, there was no significant difference in *E*. *coli* carbon source or population size in monoculture and co-culture (p = 0.43, **S2 Fig**), yet *E*. *coli* evolved resistance more slowly in co-culture. While the *S*. *enterica* carbon was more variable and its population was smaller in co-culture, the fact that both species adapted more slowly in co-culture argues against carbon source or population size being the sole drivers of the difference in evolutionary rate. Additionally, our mathematical model demonstrates that interdependency is sufficient to drive differences in rates of adaptation to abiotic stressors. In the model resources are abstracted and population sizes of each species were set to be equal in monoculture and co-culture, thereby demonstrating that differences in rate should be expected as a result of the species interactions independent of other effects.

Obligate cross-feeding also changed the mechanisms through which resistance evolved, in some cases. While in the rifampicin experiments our results were consistent with similar adaptive trajectories in monoculture and coculture, in ampicillin we saw several cases of genes repeatedly acquiring adaptive mutations in one treatment but not in the other. This was most notable for the *ftsI* mutations in *S*. *enterica*. In this case the difference in evolutionary trajectories between treatments was driven by environmentally-dependant costs. Mutations in *ftsI* repeatedly arose in co-culture replicates, but were never observed in *S*. *enterica* monocultures. While the observed *ftsI* mutations were viable in co-culture, they were lethal in monoculture. This is particularly interesting as *ftsI* activity is implicated in interactions between *S*. *enterica* and the human immune system; knocking out *ftsI* increases survival of *S*. *enterica* inside macrophage cells [35]. In previous work, acidic conditions were found to drive survival of *ftsI* knockouts [35]. We find a similar phenotypic response to acid in our mutants, though we do not believe that pH is the mechanism allowing growth of the mutants in our co-culture as we do not observe any change in the pH of our media over the course of co-culture growth. One other factor that may contribute to the different mechanisms of resistance evolution in *S*. *enterica* is carbon source. In co-culture *S*. *enterica* consumes acetate and other carbon sources while in monoculture they were provided glucose. However, *ftsI* mutants did not grow on acetate minimal medium so we do not believe that acetate is sufficient to drive the observed differences in mechanism. In future work we will further investigate the mechanisms through which our bacterial interactions alter the evolution of a gene involved in interactions with the human immune system. Our current results demonstrate that changing the costs of resistance mutations is at least one way through which co-culture can alter how bacteria evolve resistance to antibiotics.

Obligate cross-feeding also changed the mechanisms through which resistance evolved, in some cases. While in the rifampicin experiments our results were consistent with similar adaptive trajectories in monoculture and coculture, in ampicillin we saw several cases of genes repeatedly acquiring adaptive mutations in one treatment but not in the other. This was most notable for the *ftsI* mutations in *S*. *enterica*. In this case the difference in evolutionary trajectories between treatments was driven by environmentally-dependant costs. Mutations in *ftsI* repeatedly arose in co-culture replicates, but were never observed in *S*. *enterica* monocultures. While the observed *ftsI* mutations were viable in co-culture, they were lethal in monoculture. This is particularly interesting as *ftsI* activity is implicated in interactions between *S*. *enterica* and the human immune system; knocking out *ftsI* increases survival of *S*. *enterica* inside macrophage cells [35]. Our results indicate that co-culture can alter evolution by altering the cost of some resistance mechanisms, and that this change in cost can lead to changes in genes involved in interactions with the human immune system.

It is less clear why growth in co-culture altered the cost of mutations in *ftsI*. One possibility is that co-culture provided access to different carbon sources. *S*. *enterica* consumes acetate and other carbon sources in co-culture and glucose in monoculture. However, *ftsI* mutants did not grow on acetate minimal medium so we do not believe that acetate is sufficient to drive the observed differences in mechanism. In previous work, acidic conditions were found to drive survival of *ftsI* knockouts [35]. We find a similar phenotypic response to acid in our mutants, though we do not believe that pH is the mechanism allowing growth of the mutants in our co-culture either as we do not observe any change in the pH of our media over the course of co-culture growth. Though we do not suspect pH is the driver in our system a growing body of literature implicates pH as a modulator of microbial community dynamics including relative growth rates [39,40], interspecies interactions [41], and antibiotic resistance [42–44]. It is likely that a similar environmental modification alters the cost of *ftsI* mutations in our system, however future work will be needed to uncover the physiological mechanism.

Our computational model suggests an additional reason mutualism may broadly alter the mechanisms through which organisms evolve resistance to abiotic stress. Our model abstracts the specific genetic mechanisms of our system, and instead focuses on how mutualism alters selection on mutations with different effect sizes. In agreement with previous work [45,46], when large-effect resistance mutations were infrequent, evolution in monoculture simulations was driven by the mutations that caused the biggest MIC impact, despite their rarity. In contrast, in co-culture, resistance evolution occurred primarily through mutations of smaller effect. This outcome makes sense as there is little benefit to evolving resistance beyond that of obligate partners. If small effect mutations are more common and provide the same benefit as big effect mutations, then small effect mutations are more likely to be observed in evolutionary trajectories. We do not observe evidence for this effect in our current experiments likely due to the limits of our sample sizes. As noted above in our experiments, different mechanisms of resistance appear to be driven by changes in environment-dependent costs associated with specific mutations. However, the model demonstrates a broadly applicable mechanism that can lead populations involved in obligate mutualisms to evolve along different evolutionary trajectories than independent populations.

Though it was not considered here, cross-protection is also likely to alter evolution of resistance in microbial communities. Cross-protective resistance commonly arises through antibiotic degradation [21,22,47–52], though other mechanisms such as chemical signalling [23,24,53] and spatial coordination [54] have also been implicated. Neither our *E*. *coli* nor *S*. *enterica* have β-lactamases or other enzymes that degrade antibiotics, so we did not expect or observe this mechanism in our system. However, in cases where cross-protection is mutationally accessible, we would expect that MIC in co-culture could increase more quickly than in monocultures, as a single resistance mutation would protect all species in the community. There are likely many mechanisms through which bacterial interactions can alter evolution of resistance. We hope that the subset that we have demonstrated here will provide a starting point for further exploration.

The observation that mutualism changed the rate and mechanisms of adaptation to abiotic stress has several significant implications. First, the work may inform attempts to combat the crisis of antibiotic resistance. For example, treatment with broad-spectrum antibiotics that inhibit both a pathogen and its obligate cross-feeding partners could slow the rate at which pathogens acquire resistance. More broadly the work provides data on the ongoing debate over how mutualisms will respond to our rapidly changing climate [55,56]. Our results suggest that organisms involved in obligate mutualisms, such as plant-pollinator interactions, may be constrained in their ability to adapt to abiotic stress. Microbial communities provide tractable systems for developing and testing predictive frameworks for the co-evolution of mutualisms. Overall, our work emphasizes the need to take ecological context (and particularly partner resistance) into account when studying the rates and mechanisms through which populations adapt to abiotic challenges.

## Materials and methods

### Bacterial strains and media

The *Escherichia coli* and *Salmonella enterica* strains used in this study have been described previously [57]. Briefly, the *E*. *coli* str. K12 was the Δ*metB* strain from the Keio collection [58] that was mated with an hfr line to reinsert the lac operon (29). The *S*. *enterica* LT2 mutant was selected and engineered to excrete methionine [59]. Each strain is fluorescently labelled with a genomic integration of a fluorophore; *E*. *coli* is labelled with cyan fluorescent protein (CFP) and *S*. *enterica* is labelled with yellow fluorescent protein (YFP). Bacteria were grown in minimal Hypho medium containing phosphate and varying amounts and types of carbon and nitrogen as previously described [57]. Co-culture media contain 2.78mM of lactose, *E*. *coli* monoculture medium 2.78mM of lactose and 0.020mM of methionine, and *S*. *enterica* medium contained 5.55mM of glucose.

### Experimental evolution

Three different culture conditions (*E*. *coli* monoculture, *S*. *enterica* monoculture, and obligate co-culture) were each evolved across two antibiotic gradients (rifampicin, Chem-Impex International Inc. 00260, and ampicillin, Fisher BP1760). Six replicate populations were evolved for each antibiotic- culture condition combination. Each antibiotic gradient began with an antibiotic-free control well and increased twofold with each subsequent well, starting at 0.25μg/mL and ending at 64μg/mL. Stocks of antibiotics were prepared at the beginning of the experiment such that 2μL could be added to 198μL of fresh growth medium and cells to produce the desired antibiotic gradient. Monocultures and co-cultures were exposed to the same antibiotic gradients throughout the experiment. When populations evolved to grow at 64μg/mL, the upper end of the gradient was increased to 1024μg/mL and the lowest concentrations were removed. Fresh antibiotic stocks were prepared and filter-sterilized immediately before the experiment began.

Initial bacterial cultures were inoculated from DMSO freezer stocks in 10mL of monoculture minimal Hypho medium for approximately 48 hours at 30°C, to stationary phase (OD~0.4). Cells were then distributed into 96 well cell culture plates. For monocultures, 2μl of bacterial cells (~2x10^5^ cells) were inoculated into 196μl of the appropriate monoculture medium with 2μL of antibiotic stock that would confer the correct gradient concentration. In co-cultures plates, 1μl of *E*. *coli* and 1μl of *S*. *enterica* were inoculated into 196μL medium. By inoculating 2μL cells total into the growth medium for both monocultures and co-cultures, we were able to maintain starting cell densities in both cases and avoid inoculation effects. The plates were then incubated for 48 hours at 30°C with shaking at 450 rpm. After each growth phase, cells were then transferred to a new 96-well plate with fresh media. 1μL culture from wells of each antibiotic concentration were transferred to a new well with the same antibiotic concentration, and 1μL cells were transferred to a new well that was one step higher on the antibiotic gradient (see **Fig 1**). This transfer occurred whether or not there was visible growth. This regime challenged bacterial populations with double the antibiotic concentration that they were exposed to at the previous transfer, but also allowed for populations to be maintained throughout the experiment if resistance was not acquired during a given transfer. Lack of adaptation within a transfer did occur over the course of the experiment, as evidenced by any point where MIC did not increase between transfers. Additionally, by consistently inoculating 2μl total of cells into fresh media, we were able to avoid any inoculum effects [44,60] that might have altered antibiotic tolerance in a given passage. The 96-well plate from each growth phase was frozen down in 10% DMSO for future analysis. After each 48-hour growth period, each plate was also placed onto a Tecan InfinitePro 200 plate reader where OD600 and species-specific fluorescence measurements were obtained. These readings were used to calculate the 90% minimum inhibitory concentration (MIC_90_) for each replicate; growth at a given antibiotic concentration was confirmed if the well OD600 was above 10% of the OD600 of the antibiotic-free control well for a given replicate. Statistical analysis of rate of resistance evolution between monocultures and obligate co-cultures for each antibiotic was performed in R. A linear mixed- effects model with a random slope for each replicate within a treatment group was used to test the relationship between MIC and transfer, culture type (monoculture or co-culture), and a transfer-culture type interaction.

### Sequencing

To identify mutations which conferred antibiotic resistance in each evolved population, the most resistant population of each replicate in each antibiotic-growth condition combination was whole-genome sequenced. Two antibiotic-free populations per antibiotic-growth condition combination were also sequenced to identify any mutations which may have arisen during passaging but are not related to antibiotic resistance. Each population to be sequenced was scraped from 10% DMSO freezer stock and grown up in 10mL Hypho at the appropriate antibiotic concentration for 48 hours at 30°C. gDNA was then extracted from each population using Zymo Quick-gDNA Miniprep Kit (11-317C). The gDNA was then used to prepare Illumina sequencing libraries according to the Nextera XT DNA Library Prep Kit protocol. Libraries were submitted to the University of Minnesota Genomics Center for QC analysis and sequenced on an Illumina Hi-Seq with 125bp paired-end reads.

Sequence analysis was performed using BreSeq [61] to align Illumina reads to reference *E*. *coli* and *S*. *enterica* genomes as previously described [62]. Briefly, mutation lists for resistant populations were filtered such that variation between our ancestral strains and the reference genome were removed, as well as any mutations which also arose in the antibiotic-free populations. Mutation lists were then assembled for each population (**S1 Table**) and any known functions described (**S2 Table**). Mutations which rose to above 50% in frequency were examined further for their possible role in conferring antibiotic resistance (**S1 Table**).

### Isolation and phenotyping of isolates

At the final transfer of each evolution experiment, the well in each replicate displaying growth at the highest antibiotic concentration, according the MIC_90_, was identified as the ‘resistant population’. These resistant populations, as well as the corresponding antibiotic-free control populations from each replicate, were plated onto minimal Hypho medium containing X-Gal (5-Bromo-4-chloro-3-indolyl β-D-galactopyranoside, Teknova X1220), which allowed us to differentiate between β-galactosidase-positive *E*. *coli* and β-galactosidase-negative *S*. *enterica* through a colorimetric change. Three isolates of each species each from the resistant and antibiotic-free wells were selected from these plates and frozen down in 10% DMSO for further analysis. We did not genotype individual isolates, as the frequencies of most mutations of interest were sufficiently high that the isolate genotypes should reflect that of the population (**S1 Table**). The same three isolates from each population were then used to assess growth rate, yield, and MIC differences associated with specific population-level mutations.

For ampicillin-evolved populations in which we were unable to obtain *S*. *enterica* isolates on pH neutral medium, we prepared acidic Hypho plates by adding 6M HCl to the growth medium until the pH reached ~4.7. Growth medium was then autoclaved and X-gal was added as described above. For growth of these isolates in liquid medium, 6M HCl was added to autoclaved Hypho medium to a pH of ~4.7.

Evolved isolates were scraped from DMSO freezer stocks onto solid agar plates and grown up overnight, then inoculated into 96- well plates containing the appropriate species-specific Hypho and grown at 30°C for 48 hours at 450rpm to acclimatize them to minimal media growth and standardize cell density. 2μL of stationary-phase cells was then used to inoculate a new 96-well plate containing 198μL monoculture growth medium. These plates were then placed in a Tecan InfinitePro 200 for 48 hours at 30°C with shaking at 300rpm; OD600, CFP, and YFP were measured every 20 minutes. After 48 hours, 1μL from the wells of monoculture isolate plates were transferred to new 96-well plates. These plates contained 198μL of fresh co-culture Hypho medium and 1μL of either ancestor strain of *E*. *coli* or *S*. *enterica*. If the monoculture isolate was *E*. *coli*, the ancestor strain of *S*. *enterica* was added, and vice-versa. The plates were then placed into a Tecan InfinitePro 200 for 48 hours at 30°C, and OD600 and florescent data was obtained every 20 minutes. Growth rates and yields were then calculated using Baranyi curves in R software using an in-house script. Statistical analyses and graphs were prepared in Stata version 14.

Evolved isolates were prepared for inoculation as described above for beginning monoculture growth rate experiments. Based on the MIC_90_ of the population from which they originated, 2μL of each isolate culture was then inoculated across an antibiotic gradient such that the antibiotic concentration equaling the population MIC_90_ was in the middle of the gradient. An antibiotic-free control well was also included for each gradient. If the gradient used was insufficient to calculate MIC (e.g. cells grew at all concentrations, or at none of them), the experiment was repeated with the antibiotic gradient shifted up or down as necessary. MIC of isolates was determined using MIC_90_ as described above. The average MIC of isolates from each population can be found in **S7A Fig** (rifampicin-evolved populations) and **S7B Fig** (ampicillin-evolved populations**).** Statistical analyses and graphs were prepared in Stata version 14.

### Evolution model

We used an evolution simulation to examine the rate at which antibiotic resistance evolved as a function of the number of interdependent species and other variables. There were two time scales in this model. The “within-transfer” time scale, and the “between-transfer” time scale. Within a transfer, evolution was simulated in any given “well” using a modified haploid Wright-Fisher simulation with selection. A well was initiated with N individuals (default N = 1000) of each species. If this was the first transfer, these individuals were clones of a genotype with growth rate (s) = 1 and MIC = 0. The well had a pre-determined antibiotic concentration. Evolution occurred over a predetermined number of generations. Each generation, the population was fully replaced. The new population was picked from the previous generation depending on the frequency of each genotype and its growth rate. The unscaled fitness of individual *i* was determined by $w_{i}=s_{i}/\sum_{j=1}^{N}s_{j}$. This was scaled to get expected frequency $W_{i}=w_{i}/\sum_{j=1}^{N}w_{j}$. In other words, $\sum_{j=1}^{N}W_{j}=1$. The next generation was then created by generating N random numbers from a uniform distribution on [0,1] and choosing genotypes from the previous generation based on their *W_j_*. Prior to calculating *w*, each genotype’s MIC was compared to the antibiotic concentration in the well. If the genotype’s MIC was less than the antibiotic concentration, its *s* = 0 for the fitness calculation.

Once the new population was generated, some of the new individuals may be mutated. Each simulation used a pre-determined mutation rate *u* (default = 0.001 mutations per individual per generation). We generated N random numbers from a uniform distribution on [0,1]. Any random numbers less than 0.5 meant those individuals gained a mutation. In all cases, mutations could be either a growth rate mutation or an MIC mutation with a 50:50 chance. Growth rate mutations were simulated by adding a random number from a normal distribution with mean 0 and standard deviation 0.1 to the current growth rate (i.e. deleterious growth rate mutations were equally as likely as advantageous). MIC mutations altered the MIC of the individual, as described in the results. We tracked the genealogy of all individuals.

To simulate interdependence, we simulated >1 species per well. Each species had N individuals, and the dynamics of each species were independent of the other species, *except* that there must be individuals of all species with an MIC greater than the antibiotic concentration for any species to grow.

The second time scale—the transfer scale—was simulated to approximate the wet-lab experiments. Individuals were transferred from one well to two wells: the same antibiotic concentration, and the next higher antibiotic concentration. Therefore, wells received individuals from two wells. If there were surviving species in both source wells, N/2 individuals were randomly chosen from each well to populate the new well. If there were surviving species in only one source well, all N individuals were transferred from that well to the new well. If no source wells had surviving species, the destination well was sterile. Simulations were conducted in R.

Statistical analyses of the simulation data: We analyzed the rate of evolution of antibiotic tolerance as was done in the wet-lab experiments. A linear mixed- effects model with a random slope for each replicate within a treatment group was used to test the relationship between the response variable MIC and explanatory variables transfer, culture type (monoculture or co-culture), and a transfer-culture type interaction. A one-way analysis of variance tested the amount of mutations observed in the most-tolerant population, with an independent variable of the # of interdependent species. To calculate the relative tolerance of two-species simulations versus one-species simulations (**Fig 5C**), we first found the mean and standard error in the tolerance in each set of simulations. Then, the mean of the two-species community was divided by the mean of the one-species community. The error was propagated using (mean_relative_tolerance)*sqrt((SEM[two species]/mean_tolerance[two_species])^2 + (SEM[one species]/mean_tolerance[one_species])^2). The average MIC mutation size (**Fig 5D**) was the average MIC in a surviving population divided by the average number of mutations in that population. In all cases, since species were functionally equivalent, we arbitrarily chose one species to assess. Statistics were performed in R. Example code to run simulations is available as supplementary files **(S1 Code)**.

## Supporting information

S1 Table List of mutations observed following experimental evolution. (DOCX)

S2 Table Functions associated with mutated genes. (DOCX)

S1 Fig The yield of bacteria initially and after 20 transfers in the absence of antibiotic. OD equivalent is calculated from the fluorescent protein in each strain, allowing calculations of yield in the co-culture. Error bars represent standard deviations.
(PDF)

S2 Fig MICs evolved isolates containing different mutations. **A.** MICs of monoculture- and co-culture- evolved *E*. *coli* isolates containing wild-type or mutant *prc*. **B.** Monoculture and coculture growth rates of monoculture-evolved *E*. *coli* isolates containing wild-type or mutant *prc*. **C.** Monoculture and coculture growth rates of co-culture-evolved *E*. *coli* isolates containing wild-type or mutant *prc*. All p-values based on Mann-Whitney U tests. Each data point represents the average MIC for three isolates obtained from a single population. For each species- culture type combination, there are six populations total, and the statistical comparisons represent MIC comparisons between populations with wild type vs. mutant alleles.
(PDF)

S3 Fig Impact of *mdoG* and *mdoH* mutations on MICs and growth rates in rifampicin-resistant evolved *E*. *coli* and *S*. *enterica*. Each data point represents the average MIC for three isolates obtained from a single population. For each species- culture type combination, there are six populations total, and the statistical comparisons represent MIC comparisons between populations with wild type vs. mutant alleles. **A.** MIC of *mdoG* wild-type vs. mutant *E*. *coli* isolates. **B.** Monoculture and co-culture growth rates of *mdoG* wild-type vs. mutant *E*. *coli* isolates. **C.** MIC of *mdoH* wild-type vs. mutant *E*. *coli* isolates. **D.** Monoculture and co-culture growth rates of *mdoH* wild-type vs. mutant *E*. *coli* isolates. E. MIC of *mdoH* wild-type vs. mutant *S*. *enterica* isolates. **F.** Monoculture and co-culture growth rates of *mdoH* wild-type vs. mutant *S*. *enterica* isolates.
(PDF)

S4 Fig Effect of other mutations on MICs of ampicillin-evolved isolates. Each data point represents the average MIC for three isolates obtained from a single population. For each species- culture type combination, there are six populations total, and the statistical comparisons represent MIC comparisons between populations with wild type vs. mutant alleles. **A.** MICs of wild-type vs. mutant *E*. *coli* with mutations in *rne* (monoculture-evolved, in blue) and *proQ* (coculture-evolved, in green). **B.** MICs of wild-type vs. mutant *S*. *enterica* with mutations in *acrB/ompR*, *ramR*, (all evolved in monoculture only), and *metL* (evolved in co-culture only). **C.** MICs of wild-type vs. mutant *S*. *enterica* with mutations in *ompF* evolved in monoculture (gold) or co-culture (green). **D.** MICs of wild-type vs. mutant *S*. *enterica* with mutations in *yoaE* evolved in monoculture (gold) or co-culture (green).
(PDF)

S5 Fig Monoculture and co-culture growth rates of *ftsI* mutant isolates in pH = 4.7 growth medium. P = 0.0614 for monocultures, p = 0.3545 for co-cultures, Mann-Whitney U test. Each data point represents the average MIC for three isolates obtained from a single population. For each culture type combination, there are six populations total, and the statistical comparisons represent MIC comparisons between populations with wild type vs. mutant alleles.
(PDF)

S6 Fig Average number of mutations that were observed in simulations with increasing numbers of species. Error bars represent standard deviation.
(PDF)

S7 Fig Average MIC of isolates from each evolved rifampicin (**A**) and ampicillin (**B**) population. Numbers next to the data points represent population numbers. High-frequency (>50%) genotypes can be found in **S1 Table**. P-values represent Mann-Whitney U test results.
(PDF)

S1 Code A zipped file with R code that was used for simulations of antibiotic resistance evolution. (ZIP)
