# Supplementary material for: Cross-feeding modulates the rate and mechanism of antibiotic resistance evolution in a model microbial community of Escherichia coli and Salmonella enterica
Source: PLoS Pathog. 2020 Jul 20;16(7):e1008700. doi: 10.1371/journal.ppat.1008700 (PMC7392344; doi:10.1371/journal.ppat.1008700)
Supplement: S1 Table — (DOCX) [file ppat.1008700.s001.docx]

**S1 table.** List of mutations observed following experimental evolution.

| **Antibiotic** | **Evolution condition** | **Replicate** | **Library** | **Species** | **Gene** | **Description** | **Mutation** | **Position** | **Mutation Frequency** |
| --- | --- | --- | --- | --- | --- | --- | --- | --- | --- |
| Rifampicin | *E. coli* monoculture | 1 | BA010 | *E. coli* | *mdoH →* | glucosyltransferase | +T | 1,107,543:1 | 94.10% |
| Rifampicin | *E. coli* monoculture | 1 | BA010 | *E. coli* | *prc ←* | tail‑specific protease | Δ2 bp | 1,908,859 | 100% |
| Rifampicin | *E. coli* monoculture | 1 | BA010 | *E. coli* | *rpoB →* | DNA‑directed RNA polymerase subunit beta | A→C | 4,172,886 | 93.90% |
| Rifampicin | *E. coli* monoculture | 2 | BA017 | *E. coli* | *rpoB →* | DNA‑directed RNA polymerase subunit beta | C→T | 4,172,863 | 100% |
| Rifampicin | *E. coli* monoculture | 3 | BA030 | *E. coli* | *mdoG →* | glucan biosynthesis protein G mdoG → | +TT | 1,106,042:1 | 100% |
| Rifampicin | *E. coli* monoculture | 3 | BA030 | *E. coli* | *rpoB →* | DNA‑directed RNA polymerase subunit beta | G→A | 4,172,781 | 100% |
| Rifampicin | *E. coli* monoculture | 4 | BA036 | *E. coli* | *glpA →* | anaerobic glycerol‑3‑phosphate dehydrogenase subunit A | +CTGCGCGGG | 2,346,218 | 100% |
| Rifampicin | *E. coli* monoculture | 4 | BA036 | *E. coli* | *mdoG →* | glucan biosynthesis protein G mdoG → | G→C | 1,105,310 | 64.80% |
| Rifampicin | *E. coli* monoculture | 4 | BA036 | *E. coli* | *prc ←* | tail‑specific protease | Δ11 bp | 1,907,811 | 100% |
| Rifampicin | *E. coli* monoculture | 5 | BA049 | *E. coli* | *mdoG →* | glucan biosynthesis protein G mdoG → | Δ8 bp | 1,105,573 | 100% |
| Rifampicin | *E. coli* monoculture | 5 | BA049 | *E. coli* | *rpoB →* | DNA‑directed RNA polymerase subunit beta | A→G | 4,172,719 | 100% |
| Rifampicin | *E. coli* monoculture | 6 | BA056 | *E. coli* | *fre →* | NAD(P)H‑flavin reductase | Δ13 bp | 4,019,988 | 86.50% |
| Rifampicin | *E. coli* monoculture | 6 | BA056 | *E. coli* | *mdoH →* | glucosyltransferase | Δ6 bp | 1,107,321 | 100% |
| Rifampicin | *S. enterica* monoculture | 1 | BA067 | *S. enterica* | *mdoH →* | gucans biosynthesis glucosyltransferase H | G→T | 1,239,272 | 100% |
| Rifampicin | *S. enterica* monoculture | 1 | BA067 | *S. enterica* | *rpoB →* | DNA‑directed RNA polymerase subunit beta | T→G | 4,367,622 | 100% |
| Rifampicin | *S. enterica* monoculture | 2 | BA079 | *S. enterica* | *ispD ←* | 2‑C‑methyl‑D‑erythritol 4‑phosphate cytidylyltransferase | C→T | 3,070,986 | 100% |
| Rifampicin | *S. enterica* monoculture | 2 | BA079 | *S. enterica* | *mdoH →* | gucans biosynthesis glucosyltransferase H | G→T | 1,239,272 | 100% |
| Rifampicin | *S. enterica* monoculture | 2 | BA079 | *S. enterica* | *rpoB →* | DNA‑directed RNA polymerase subunit beta | A→G | 4,367,454 | 100% |
| Rifampicin | *S. enterica* monoculture | 2 | BA079 | *S. enterica* | *STM4466 ←* | carbamate kinase | A→G | 4,708,815 | 100% |
| Rifampicin | *S. enterica* monoculture | 4 | BA098 | *S. enterica* | *mdoH →* | gucans biosynthesis glucosyltransferase H | G→T | 1,239,272 | 100% |
| Rifampicin | *S. enterica* monoculture | 4 | BA098 | *S. enterica* | *rpoB →* | DNA‑directed RNA polymerase subunit beta | C→T | 4,367,442 | 100% |
| Rifampicin | *S. enterica* monoculture | 5 | BA108 | *S. enterica* | *rpoB →* | DNA‑directed RNA polymerase subunit beta | C→T | 4,367,511 | 90.00% |
| Rifampicin | *S. enterica* monoculture | 5 | BA108 | *S. enterica* | *rpoB →* | DNA‑directed RNA polymerase subunit beta | T→G | 4,367,622 | 100% |
| Rifampicin | *S. enterica* monoculture | 5 | BA108 | *S. enterica* | *ramR ←* | regulatory protein | Δ4 bp | 638,200 | 86.10% |
| Rifampicin | *S. enterica* monoculture | 6 | BA115 | *S. enterica* | *mdoH →* | glucans biosynthesis glucosyltransferase H | G→T | 1,239,272 | 100% |
| Rifampicin | *S. enterica* monoculture | 6 | BA115 | *S. enterica* | *rpoB →* | DNA‑directed RNA polymerase subunit beta | C→T | 4,367,504 | 100% |
| Rifampicin | Co-culture | 1 | BA125 | *E. coli* | *pnp ←* | polyribonucleotide nucleotidyltransferase | +G | 3,303,449:1 | 90.10% |
| Rifampicin | Co-culture | 1 | BA125 | *E. coli* | *rpoB →* | DNA‑directed RNA polymerase subunit betat | T→A | 4,172,887 | 100% |
| Rifampicin | Co-culture | 2 | BA139 | *E. coli* | *rfaQ ←* | LPS core heptosyltransferase RfaQ | Δ1 bp | 3,800,489 | 100% |
| Rifampicin | Co-culture | 2 | BA139 | *E. coli* | *rpoB →* | DNA‑directed RNA polymerase subunit beta | C→A | 4,172,893 | 100% |
| Rifampicin | Co-culture | 2 | BA139 | *S. enterica* | *rpoB →* | DNA‑directed RNA polymerase subunit beta | A→G | 4,366,350 | 100% |
| Rifampicin | Co-culture | 3 | BA145 | *E. coli* | *prc ←* | tail‑specific protease | Δ2 bp | 1,908,859 | 100% |
| Rifampicin | Co-culture | 3 | BA145 | *E. coli* | *prs ←* | ribose‑phosphate pyrophosphokinase | A→T | 1,256,880 | 100% |
| Rifampicin | Co-culture | 3 | BA145 | *E. coli* | *rpoB →* | DNA‑directed RNA polymerase subunit beta | C→A | 4,172,893 | 100% |
| Rifampicin | Co-culture | 3 | BA145 | *S. enterica* | *mdoH →* | glucans biosynthesis glucosyltransferase H | Δ6 bp | 1,239,767 | 22.2% |
| Rifampicin | Co-culture | 4 | BA155 | *E. coli* | *prc ←* | tail‑specific protease | Δ10 bp | 1,907,831 | 64.40% |
| Rifampicin | Co-culture | 5 | BA165 | *E. coli* | *rplK →* | 50S ribosomal protein L11 | C→T | 4,168,451 | 100% |
| Rifampicin | Co-culture | 5 | BA165 | *E. coli* | *rpoB →* | DNA‑directed RNA polymerase subunit beta | C→A | 4,172,893 | 92.80% |
| Rifampicin | Co-culture | 5 | BA165 | *E. coli* | *BW25113_RS13710* ←  / ← *BW25113_RS13715* | CP4‑57 defective prophage, DUF4297/DUF1837; polymorphic toxin family protein/hypothetical protein | +GCACTATG | 2,758,778 | 87.60% |
| Rifampicin | Co-culture1 | 5 | BA165 | *S. enterica* | *mdoH →* | glucans biosynthesis glucosyltransferase H | C→T | 1,238,069 | 58.7% |
| Rifampicin | Co-culture | 6 | BA176 | *E. coli* | *prc ←* | tail‑specific protease | Δ11 bp | 1,908,656 | 100% |
| Rifampicin | Co-culture | 6 | BA176 | *S. enterica* | *rpoB →* | DNA‑directed RNA polymerase subunit beta | C→A | 4,367,483 | 100% |
| Ampicillin | *E. coli* monoculture | 1 | rMM010 | *E. coli* | *acrB ←* | multidrug efflux RND transporter permease subunit | A→C | 479,480 | 100% |
| Ampicillin | *E. coli* monoculture | 1 | rMM010 | *E. coli* | *rne ←* | ribonuclease E | repeat_region (+) +5 bp :: Δ1 bp | 1,138,341 | 100% |
| Ampicillin | *E. coli* monoculture | 2 | rMM020 | *E. coli* | *envZ ←* | two‑component sensor histidine kinase | C→G | 3,528,288 | 100% |
| Ampicillin | *E. coli* monoculture | 4 | rMM039 | *E. coli* | *mdoH →* | glucosyltransferase | Δ1 bp | 1,107,469 | 100% |
| Ampicillin | *E. coli* monoculture | 5 | rMM049 | *E. coli* | *ilvN ←* | acetolactate synthase isozyme 1 small subunit | Δ5 bp | 3,844,420 | 72.20% |
| Ampicillin | *E. coli* monoculture | 6 | rMM060 | *E. coli* | *eda ←* | 2‑keto‑3‑deoxy‑L‑rhamnonate aldolase | Δ47 bp | 2,351,941 | 67.60% |
| Ampicillin | *E. coli* monoculture | 6 | rMM060 | *E. coli* | *ompF ←* | outer membrane protein F | Δ2 bp | 982,235 | 100% |
| Ampicillin | *E. coli* monoculture | 6 | rMM060 | *E. coli* | *prlF →* | antitoxin PrlF | repeat_region (–) +4 bp :: Δ3 bp | 3,270,368 | 59.20% |
| Ampicillin | *E. coli* monoculture | 6 | rMM060 | *E. coli* | *rne ←* | ribonuclease E | repeat_region (+) +5 bp :: Δ1 bp | 1,138,341 | 100% |
| Ampicillin | *S. enterica* monoculture | 1 | rMM067 | *S. enterica* | *ompF/IS10* | outer membrane protein F/ repeat region | IS element insertion | 1090025 =  = 1090033 | 85% |
| Ampicillin | *S. enterica* monoculture | 2 | rMM078 | *S. enterica* | *ompF/IS10* | outer membrane protein F/ repeat region | IS element insertion | 1090025 =  = 1090033 | 95% |
| Ampicillin | *S. enterica* monoculture | 2 | rMM078 | *S. enterica* | *ramR ←* | regulatory protein | coding (511‑554/582 nt) | 638,174 | 100% |
| Ampicillin | *S. enterica* monoculture | 3 | rMM090 | *S. enterica* | *acrB ←* | RND family acridine efflux pump | W634R (TGG→CGG) | 530,497 | 100% |
| Ampicillin | *S. enterica* monoculture | 3 | rMM090 | *S. enterica* | *ompR ←* | osmolarity response regulator OmpR | R210L (CGT→CTT) | 3,659,697 | 100% |
| Ampicillin | *S. enterica* monoculture | 3 | rMM090 | *S. enterica* | *ramR ←* | regulatory protein | Q19* (CAG→TAG) | 638,673 | 100% |
| Ampicillin | *S. enterica* monoculture | 3 | rMM090 | *S. enterica* | *yoaE* | inner membrane protein | Intragenic inversion | = 1926896 | 100% |
| Ampicillin | *S. enterica* monoculture | 5 | rMM108 | *S. enterica* | *ompF/IS10* | outer membrane protein F/ repeat region | IS element insertion | 1090025 =  = 1090033 | 73% |
| Ampicillin | *S. enterica* monoculture | 5 | rMM108 | *S. enterica* | *yoaE* | inner membrane protein | Intragenic inversion | = 1926896 | 84% |
| Ampicillin | *S. enterica* monoculture | 6 | rMM119 | *S. enterica* | *acrB ←* | RND family acridine efflux pump | F615S (TTC→TCC) | 530,553 | 100% |
| Ampicillin | *S. enterica* monoculture | 6 | rMM119 | *S. enterica* | *ompR ←* | osmolarity response regulator OmpR | R210L (CGT→CTT) | 3,659,697 | 100% |
| Ampicillin | Co-culture | 1 | rMM127 | *S. enterica* | *ahpF →* | alkyl hydroperoxide reductase subunit F | G→A | 672,700 | 94.60% |
| Ampicillin | Co-culture | 1 | rMM127 | *S. enterica* | *amn ←* | AMP nucleosidase | T→C | 2,092,111 | 100% |
| Ampicillin | Co-culture | 1 | rMM127 | *S. enterica* | *dnaQ →* | DNA polymerase III subunit epsilon | T→A | 303,499 | 100% |
| Ampicillin | Co-culture | 1 | rMM127 | *S. enterica* | *envZ ←* | osmolarity sensor protein EnvZ | A→G | 3,659,359 | 100% |
| Ampicillin | Co-culture | 1 | rMM127 | *S. enterica* | *ftsI →* | peptidoglycan synthase FtsI | +A | 143,219:1 | 100% |
| Ampicillin | Co-culture | 1 | rMM127 | *S. enterica* | *ftsZ →* | cell division protein FtsZ | C→T | 155,877 | 100% |
| Ampicillin | Co-culture | 1 | rMM127 | *S. enterica* | *gldA ← / → STM3531* | glycerol dehydrogenase/  dihydroxyacid dehydratase | G→A | 3,694,118 | 94.40% |
| Ampicillin | Co-culture | 1 | rMM127 | *S. enterica* | *metL →* | bifunctional aspartate kinase II/  homoserine dehydrogenase II | G→A | 4,312,839 | 100% |
| Ampicillin | Co-culture | 1 | rMM127 | *S. enterica* | *rtn →* | lambda/N4 phages resistance membrane protein | T→C | 2,315,609 | 92.40% |
| Ampicillin | Co-culture | 1 | rMM127 | *S. enterica* | *sppA ←* | protease 4 | G→A | 1,373,495 | 54.20% |
| Ampicillin | Co-culture | 1 | rMM127 | *S. enterica* | *STM0019 →* | hydroxymethyltransferase | A→G | 20,208 | 100% |
| Ampicillin | Co-culture | 1 | rMM127 | *S. enterica* | *STM0566 →* | inner membrane protein | G→A | 622,193 | 100% |
| Ampicillin | Co-culture | 1 | rMM127 | *S. enterica* | *STM1552 → / ← STM05155* | cytoplasmic protein/hypothetical protein | A→G | 1,629,730 | 100% |
| Ampicillin | Co-culture | 1 | rMM127 | *S. enterica* | *STM2179 ←* | sugar transporter | T→C | 2,275,700 | 93.00% |
| Ampicillin | Co-culture | 1 | rMM127 | *S. enterica* | *STM2700 ←* | phage tail fiber‑like protein | T→C | 2,850,036 | 94.00% |
| Ampicillin | Co-culture | 1 | rMM127 | *S. enterica* | *STM2739 →* | phage tail‑like protein | C→A | 2,877,206 | 100% |
| Ampicillin | Co-culture | 1 | rMM127 | *S. enterica* | *STM2756 ←* | sugar phosphate aminotransferase | C→T | 2,894,787 | 100% |
| Ampicillin | Co-culture | 1 | rMM127 | *S. enterica* | *STM3052 ←* | outer membrane protein | T→C | 3,211,576 | 94.00% |
| Ampicillin | Co-culture | 1 | rMM127 | *S. enterica* | *STM3631 ←* | xanthine permease | T→C | 3,817,700 | 94.60% |
| Ampicillin | Co-culture | 1 | rMM127 | *S. enterica* | *STM3653 ← / ← glyS* | acetyltransferase/glycine‑‑tRNA ligase subunit beta | A→G | 3,839,640 | 100% |
| Ampicillin | Co-culture | 1 | rMM127 | *S. enterica* | *STM4419 →* | sugar transporter | C→T | 4,662,084 | 100% |
| Ampicillin | Co-culture | 1 | rMM127 | *S. enterica* | *xylA ← / → xylR* | xylose isomerase/xylose operon regulatory protein | T→C | 3,848,052 | 100% |
| Ampicillin | Co-culture | 1 | rMM127 | *S. enterica* | *yeaQ →* | inner membrane protein | T→C | 1,353,286 | 54.60% |
| Ampicillin | Co-culture | 1 | rMM127 | *S. enterica* | *yhiP →* | dipeptide/tripeptide permease B | C→T | 3,762,685 | 52.60% |
| Ampicillin | Co-culture | 2 | rMM137 | *E. coli* | *proQ ←* | RNA chaperone ProQ | Δ5 bp | 1,909,389 | 19.70% |
| Ampicillin | Co-culture | 2 | rMM137 | *S. enterica* | *metL →* | bifunctional aspartate kinase II/  homoserine dehydrogenase II | Δ4 bp | 4,311,847 | 59.20% |
| Ampicillin | Co-culture | 3 | rMM146 | *S. enterica* | *ompF ←* | outer membrane protein F | Δ116 bp | 1,090,110 | 64.50% |
| Ampicillin | Co-culture | 4 | rMM158 | *E. coli* | *proQ ←* | RNA chaperone ProQ | C→A | 1,909,737 | 86.50% |
| Ampicillin | Co-culture | 4 | rMM158 | *S. enterica* | *ftsI →* | peptidoglycan synthase FtsI | G→T | 143,325 | 75.30% |
| Ampicillin | Co-culture | 5 | rMM167 | *S. enterica* | *yoaE* | inner membrane protein | Intragenic inversion | = 1926896 | 100% |
