# Supplementary material for: Cross-feeding modulates the rate and mechanism of antibiotic resistance evolution in a model microbial community of Escherichia coli and Salmonella enterica
Source: PLoS Pathog. 2020 Jul 20;16(7):e1008700. doi: 10.1371/journal.ppat.1008700 (PMC7392344; doi:10.1371/journal.ppat.1008700)
Supplement: S2 Table — (DOCX) [file ppat.1008700.s002.docx]

**S2 table.** Functions associated with mutated genes.

| Gene | Mutated in | Description | Function |
| --- | --- | --- | --- |
| *fre →* | *E. coli*: BA056 | NAD(P)H‑flavin reductase | May be involved in iron homeostasis and oxidative stress response [1] |
| *rpoB →* | *E. coli*: BA010, BA017, BA030, BA049, BA125, BA139, BA145, BA165  *S. enterica*: BA067, BA079, BA098, BA108, BA115, BA139, BA176 | DNA‑directed RNA polymerase subunit beta | RNA polymerase beta subunit; commonly mutated in rifampicin-resistant lines [2] |
| *prc ←* | *E. coli*: BA010, BA036, BA145, BA155, BA176 | tail‑specific protease | Cleaves precursor to form a functional PBP3; involved in thermal and osmotic stress response [3]; mutations lead to increased antibiotic susceptibility [4] |
| *mdoH →* | *E. coli*: BA010, BA056, rMM039  *S. enterica*: BA067, BA079, BA098, BA115 | glucosyltransferase | Also called *mdoH/opgH*; involved in regulating cell wall osmolarity and likely modulates cellular penetration of rifampicin [5] |
| *mdoG →* | *E. coli*: BA030, BA036, BA049 | glucan biosynthesis protein G MdoG → | Required for the synthesis of osmoregulated periplasmic glucans; may function in biofilm formation [6] |
| *glpA →* | *E. coli*: BA036 | anaerobic glycerol‑3‑phosphate dehydrogenase subunit A | Involved in utilization of glycerol as a carbon source; deletions lead to decreased persister formation [7] |
| *pnp ←* | *E. coli*: BA125 | polyribonucleotide nucleotidyltransferase | Involved in mRNA degradation and tRNA processing; contributes to rRNA quality control during steady-state growth [8] |
| *rfaQ ←* | *E. coli*: BA139 | LPS core heptosyltransferase RfaQ | Also called *waaQ*; involved in LPS biosynthesis [1] |
| *prs ←* | *E. coli*: BA145 | ribose‑phosphate pyrophosphokinase | Involved in central metabolism [1] |
| BW25113_RS13710 ←  / ← BW25113_RS13715 | *E. coli*: BA165 | CP4‑57 defective prophage, DUF4297/DUF1837 polymorphic toxin family protein/hypothetical protein |  |
| *rplK →* | *E. coli*: BA165 | 50S ribosomal protein L11 | Regulator of the stringent response and signals increased ppGpp production, which increases antibiotic tolerance [9] |
| *rne ←* | *E. coli*: rMM010, rMM060 | ribonuclease E | Small regulatory RNA that functions in SOS initiation [10] |
| *acrB ←* | *E. coli*: rMM010  *S. enterica*: rMM090, rMM119 | multidrug efflux RND transporter permease subunit | Efflux transporter protein component of the TolC-AcrAB multidrug efflux pump [11] |
| *envZ ←* | *E. coli*: rMM020  *S. enterica*: rMM127 | two‑component sensor histidine kinase | Sensor kinase in two-component signalling control of *ompF/ompC* expression regulation [11] |
| *ilvN ←* | *E. coli*: rMM049 | acetolactate synthase isozyme 1 small subunit | Catalyzes the first step in valine biosynthesis and the second step in isoleucine biosynthesis [1] |
| *eda ←* | *E. coli*: rMM060 | 2‑keto‑3‑deoxy‑L‑rhamnonate aldolase | Involved in glucose degradation through the Entner-Doudoroff pathway [1] |
| *prlF →* | *E. coli*: rMM060 | antitoxin PrlF | Antitoxin component of an mRNA degradation toxin system [12] |
| *ompF ←* | *E. coli*: rMM060  *S. enterica*: rMM146 | outer membrane protein F | Classic trimeric porin commonly lost in beta-lactam resistant strains [13] |
| *proQ ←* | *E. coli*: rMM137, rMM158 | RNA chaperone ProQ | Small regulatory RNA that controls efflux pump expression [14] |
| *ispD ←* | *S. enterica*: BA079 | 2‑C‑methyl‑D‑erythritol 4‑phosphate cytidylyltransferase | Functions in isoprene biosynthesis [1] |
| *STM4466 ←* | *S. enterica*: BA079 | carbamate kinase | Functions in the arginine deaminase pathway [1] |
| *ramR ←* | *S. enterica*: BA108, rMM078, rMM090 | regulatory protein | Negative repressor of *ramA*, which positively regulates *acrAB* expression- knockouts result in constitutive *acrAB* expression [15] |
| *ompF/IS10* | *S. enterica*: rMM067, rMM078, rMM108 | outer membrane protein F/ repeat region | Classic trimeric porin commonly lost in beta-lactam resistant strains [13] |
| *yoaE* | *S. enterica*: rMM090, rMM108, rMM167 | inner membrane protein | Integral membrane protein, putative transporter [1] |
| *ompR ←* | *S. enterica*: rMM090, rMM119 | osmolarity response regulator OmpR | Response regulator in two-component signalling of *ompF/ompC* expression [11] |
| *ahpF →* | *S. enterica*: rMM127 | alkyl hydroperoxide reductase subunit F | Functions in protecting cells from hydrogen peroxide toxicity [16] |
| *amn ←* | *S. enterica*: rMM127 | AMP nucleosidase | Loss of function mutations allow greater cold tolerance in *E. coli* [17] |
| *dnaQ →* | *S. enterica*: rMM127 | DNA polymerase III subunit epsilon | Encodes proofreading DNA polymerase III; mutations lead to high mutation rates and are often observed under antibiotic selection [18] |
| *ftsZ →* | *S. enterica*: rMM127 | cell division protein FtsZ | Essential component of cell division (forms septum Z-ring); target of antimicrobial development [19] |
| *gldA ← / → STM3531* | *S. enterica*: rMM127 | glycerol dehydrogenase/dihydroxyacid dehydratase | - |
| *rtn →* | *S. enterica*: rMM127 | lambda/N4 phages resistance membrane protein | - |
| *sppA ←* | *S. enterica*: rMM127 | protease 4 | Signal peptide peptidase [1] |
| *STM0019 →* | *S. enterica*: rMM127 | hydroxymethyltransferase | - |
| *STM0566 →* | *S. enterica*: rMM127 | inner membrane protein | - |
| *STM1552 → / ← STM05155* | *S. enterica*: rMM127 | cytoplasmic protein/hypothetical protein | - |
| *STM2179 ←* | *S. enterica*: rMM127 | sugar transporter | 4-hydroxybenzoate transporter [1] |
| *STM2700 ←* | *S. enterica*: rMM127 | phage tail fiber‑like protein | - |
| *STM2739 →* | *S. enterica*: rMM127 | phage tail‑like protein | Putative integrase [1] |
| *STM2756 ←* | *S. enterica*: rMM127 | sugar phosphate aminotransferase | - |
| *STM3052 ←* | *S. enterica*: rMM127 | outer membrane protein | May be involved in phenol degradation [1] |
| *STM3631 ←* | *S. enterica*: rMM127 | xanthine permease | - |
| *STM3653 ← / ← glyS* | *S. enterica*: rMM127 | acetyltransferase/glycine‑‑tRNA ligase subunit beta | - |
| *STM4419 →* | *S. enterica*: rMM127 | sugar transporter | Carbohydrate/proton symporter [1] |
| *xylA ← / → xylR* | *S. enterica*: rMM127 | xylose isomerase/xylose operon regulatory protein | - |
| *yeaQ →* | *S. enterica*: rMM127 | inner membrane protein | Putative integral membrane protein [1] |
| *yhiP →* | *S. enterica*: rMM127 | dipeptide/tripeptide permease B | Putative transporter [1] |
| *metL →* | *S. enterica*: rMM127, rMM137 | bifunctional aspartate kinase II/homoserine dehydrogenase II | Catalyzes the first step in lysine/homoserine biosynthesis, the last step in homoserine biosynthesis, and indirectly functions in methionine and threonine biosynthesis [1] |
| *ftsI →* | *S. enterica*: rMM127, rMM158 | peptidoglycan synthase FtsI | Penicillin-binding protein 3 (PBP3); mutations confer β-lactam resistance [20] |

References for table S2

1. UniProt: a worldwide hub of protein knowledge. Nucleic Acids Res. 2019 Jan 8;47(D1):D506–15.

2. Campbell EA, Korzheva N, Mustaev A, Murakami K, Nair S, Goldfarb A, et al. Structural mechanism for rifampicin inhibition of bacterial RNA polymerase. Cell. 2001 Mar 23;104(6):901–12.

3. Kerr CH, Culham DE, Marom D, Wood JM. Salinity-dependent impacts of *proQ, prc,* and *spr* deficiencies on *Escherichia coli* cell structure. J Bacteriol. 2014 Mar;196(6):1286–96.

4. Seoane A, Sabbaj A, McMurry LM, Levy SB. Multiple antibiotic susceptibility associated with inactivation of the *prc* gene. J Bacteriol. 1992 Dec;174(23):7844–7.

5. Falchi FA, Maccagni EA, Puccio S, Peano C, De Castro C, Palmigiano A, et al. Mutation and suppressor analysis of the essential lipopolysaccharide transport protein LptA reveals strategies to overcome severe outer membrane permeability defects in *Escherichia coli*. J Bacteriol. 2017 Dec 20;200(2):e00487-17.

6. Hanoulle X, Rollet E, Clantin B, Landrieu I, Ödberg-Ferragut C, Lippens G, et al. Structural analysis of *Escherichia coli* OpgG, a protein required for the biosynthesis of osmoregulated periplasmic glucans. Journal of Molecular Biology. 2004 Sep 3;342(1):195–205.

7. Spoering AL, Vulić M, Lewis K. GlpD and PlsB participate in persister cell formation in *Escherichia coli*. J Bacteriol. 2006 Jul;188(14):5136–44.

8. Basturea GN, Zundel MA, Deutscher MP. Degradation of ribosomal RNA during starvation: comparison to quality control during steady-state growth and a role for RNase PH. RNA. 2011 Feb;17(2):338–45.

9. Agrawal P, Varada R, Sah S, Bhattacharyya S, Varshney U. Species-specific interactions of Arr with RplK mediate stringent response in bacteria. J Bacteriol. 2018 Feb 23;200(6):e00722-17.

10. Manasherob R, Miller C, Kim K, Cohen SN. Ribonuclease E modulation of the bacterial SOS response. PLOS ONE. 2012 Jun 8;7(6):e38426.

11. Adler M, Anjum M, Andersson DI, Sandegren L. Combinations of mutations in e*nvZ, ftsI, mrdA, acrB* and *acrR* can cause high-level carbapenem resistance in *Escherichia coli.* J Antimicrob Chemother. 2016 May;71(5):1188–98.

12. Schmidt O, Schuenemann VJ, Hand NJ, Silhavy TJ, Martin J, Lupas AN, et al. *prlF* and *yhaV* encode a new toxin-antitoxin system in *Escherichia coli*. J Mol Biol. 2007 Sep 28;372(4):894–905.

13. Nikaido H. Molecular basis of bacterial outer membrane permeability revisited. Microbiol Mol Biol Rev. 2003 Dec;67(4):593–656.

14. Dersch P, Khan MA, Mühlen S, Görke B. Roles of regulatory RNAs for antibiotic resistance in bacteria and their potential value as novel drug targets. Front Microbiol. 2017;8:803.

15. Abouzeed YM, Baucheron S, Cloeckaert A. *ramR* mutations involved in efflux-mediated multidrug resistance in *Salmonella enterica* Serovar Typhimurium. Antimicrobial Agents and Chemotherapy. 2008 Jul 1;52(7):2428–34.

16. Ling J, Cho C, Guo L-T, Aerni H, Rinehart J, Söll D. Protein aggregation caused by aminoglycoside action is prevented by a hydrogen peroxide scavenger. Mol Cell. 2012 Dec 14;48(5):713–22.

17. Parry BR, Shain DH. Manipulations of AMP metabolic genes increase growth rate and cold tolerance in *Escherichia coli:* Implications for psychrophilic evolution. Mol Biol Evol. 2011 Jul 1;28(7):2139–45.

18. Baym M, Lieberman TD, Kelsic ED, Chait R, Gross R, Yelin I, et al. Spatiotemporal microbial evolution on antibiotic landscapes. Science. 2016 Sep 9;353(6304):1147–51.

19. Fujita J, Maeda Y, Mizohata E, Inoue T, Kaul M, Parhi AK, et al. Structural flexibility of an inhibitor overcomes drug resistance mutations in *Staphylococcus aureus ftsZ*. ACS Chem Biol. 2017 Jul 21;12(7):1947–55.

20. Sun S, Selmer M, Andersson DI. Resistance to β-lactam antibiotics conferred by point mutations in penicillin-binding proteins PBP3, PBP4 and PBP6 in *Salmonella enterica.* PLoS ONE. 2014;9(5):e97202.
