# Supplementary material for: Cross-feeding modulates the rate and mechanism of antibiotic resistance evolution in a model microbial community of Escherichia coli and Salmonella enterica
Source: PLoS Pathog. 2020 Jul 20;16(7):e1008700. doi: 10.1371/journal.ppat.1008700 (PMC7392344; doi:10.1371/journal.ppat.1008700)
Supplement: S1 Fig — OD equivalent is calculated from the fluorescent protein in each strain, allowing calculations of yield in the co-culture. Error bars represent standard deviations. (PDF) [file ppat.1008700.s003.pdf]

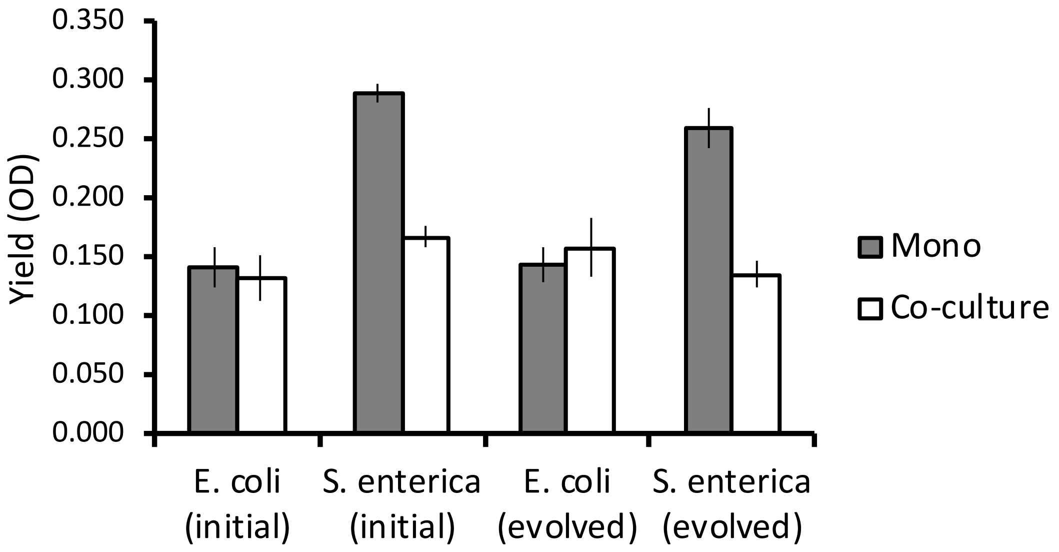

S1 fig. The yield of bacteria initially and after 20 transfers in the absence of antibiotic. OD equivalent is calculated from the fluorescent protein in each strain, allowing calculations of yield in the co-culture. Error bars represent standard deviations.
