# Supplementary material for: Cross-feeding modulates the rate and mechanism of antibiotic resistance evolution in a model microbial community of Escherichia coli and Salmonella enterica
Source: PLoS Pathog. 2020 Jul 20;16(7):e1008700. doi: 10.1371/journal.ppat.1008700 (PMC7392344; doi:10.1371/journal.ppat.1008700)
Supplement: S2 Fig — A. MICs of monoculture- and co-culture- evolved E. coli isolates containing wild-type or mutant prc. B. Monoculture and coculture growth rates of monoculture-evolved E. coli isolates containing wild-type or mutant prc. C. Monoculture and coculture growth rates of co-culture-evolved E. coli isolates containing wild-type or mutant prc. All p-values based on Mann-Whitney U tests. Each data point represents the average MIC for three isolates obtained from a single population. For each species- culture type combination, there are six populations total, and the statistical comparisons represent MIC comparisons between populations with wild type vs. mutant alleles. (PDF) [file ppat.1008700.s004.pdf]

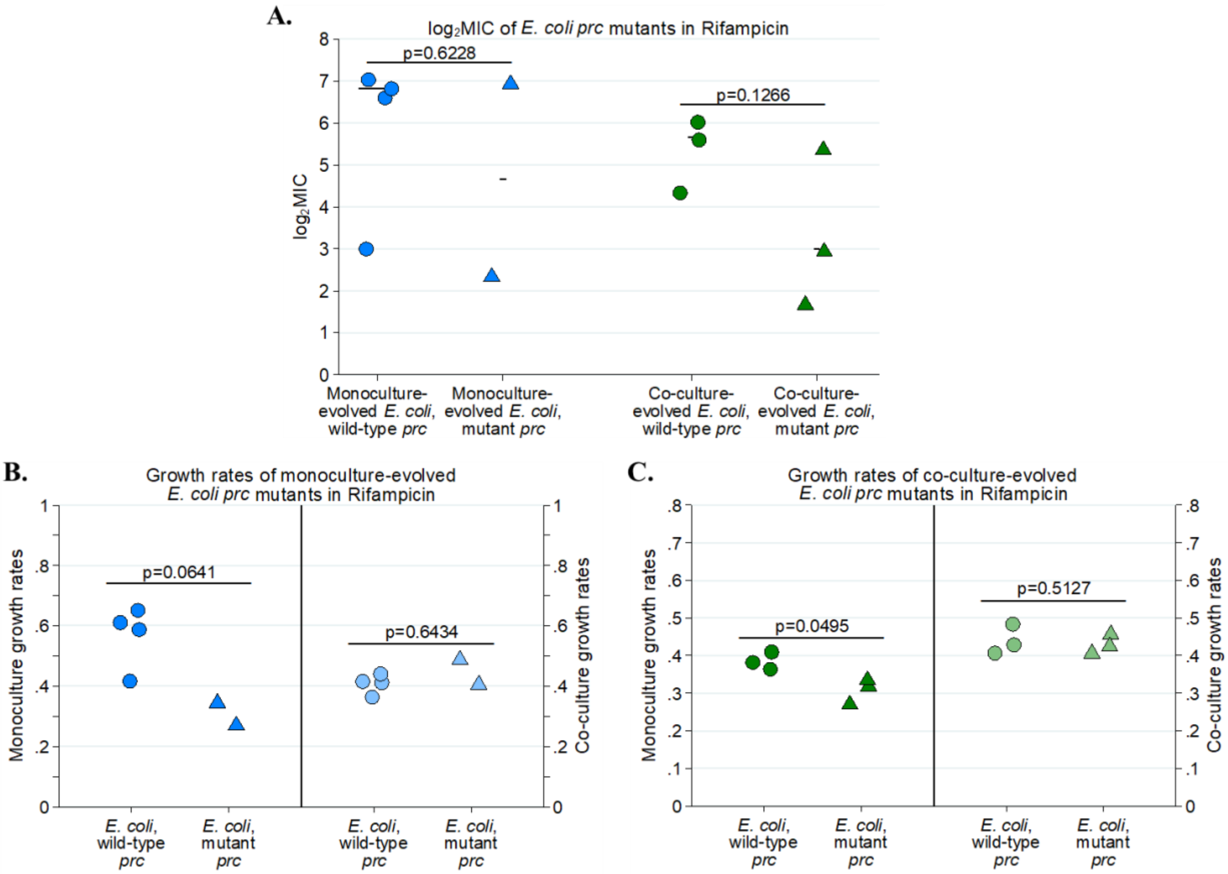

S2 fig. MICs evolved isolates containing different mutations. **A.** MICs of monoculture- and co-culture- evolved *E. coli* isolates containing wild-type or mutant *prc*. **B.** Monoculture and coculture growth rates of monoculture-evolved *E. coli* isolates containing wild-type or mutant *prc*. **C.** Monoculture and coculture growth rates of co-culture-evolved *E. coli* isolates containing wild-type or mutant *prc*. All p-values based on Mann-Whitney U tests. Each data point represents the average MIC for three isolates obtained from a single population. For each species- culture type combination, there are six populations total, and the statistical comparisons represent MIC comparisons between populations with wild type vs. mutant alleles.
