# Supplementary material for: Cross-feeding modulates the rate and mechanism of antibiotic resistance evolution in a model microbial community of Escherichia coli and Salmonella enterica
Source: PLoS Pathog. 2020 Jul 20;16(7):e1008700. doi: 10.1371/journal.ppat.1008700 (PMC7392344; doi:10.1371/journal.ppat.1008700)
Supplement: S3 Fig — Each data point represents the average MIC for three isolates obtained from a single population. For each species- culture type combination, there are six populations total, and the statistical comparisons represent MIC comparisons between populations with wild type vs. mutant alleles. A. MIC of mdoG wild-type vs. mutant E. coli isolates. B. Monoculture and co-culture growth rates of mdoG wild-type vs. mutant E. coli isolates. C. MIC of mdoH wild-type vs. mutant E. coli isolates. D. Monoculture and co-culture growth rates of mdoH wild-type vs. mutant E. coli isolates. E. MIC of mdoH wild-type vs. mutant S. enterica isolates. F. Monoculture and co-culture growth rates of mdoH wild-type vs. mutant S. enterica isolates. (PDF) [file ppat.1008700.s005.pdf]

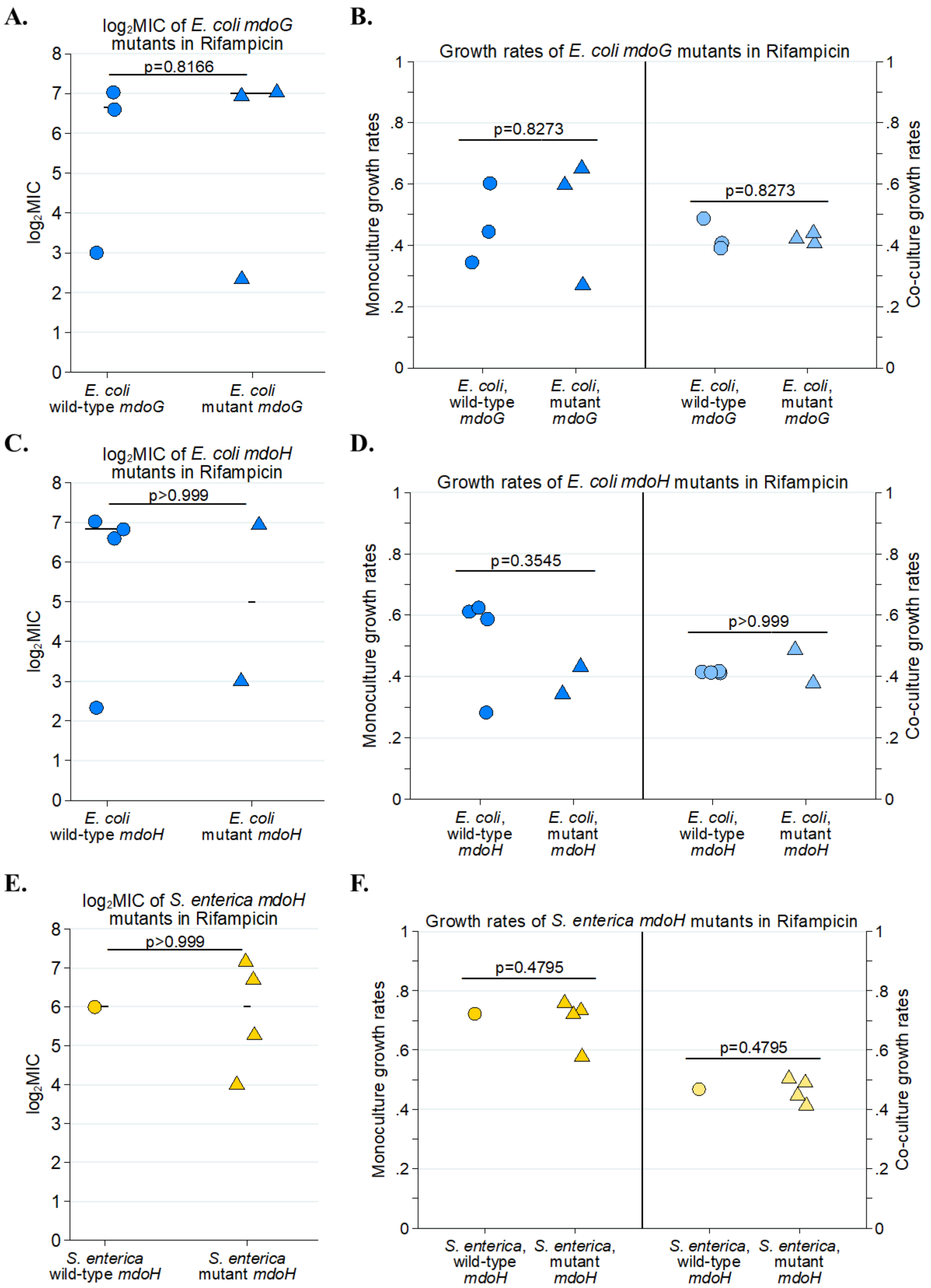
A.
log2MIC of E. coli mdoG mutants in Rifampicin
p=0.8166
log2MIC
E. coli wild-type mdoG
E. coli mutant mdoG
B.
Growth rates of E. coli mdoG mutants in Rifampicin
p=0.8273
p=0.8273
Monoculture growth rates
Co-culture growth rates
E. coli, wild-type mdoG
E. coli, mutant mdoG
E. coli, wild-type mdoG
E. coli, mutant mdoG
C.
log2MIC of E. coli mdoH mutants in Rifampicin
p>0.999
log2MIC
E. coli wild-type mdoH
E. coli mutant mdoH
D.
Growth rates of E. coli mdoH mutants in Rifampicin
p=0.3545
p>0.999
Monoculture growth rates
Co-culture growth rates
E. coli, wild-type mdoH
E. coli, mutant mdoH
E. coli, wild-type mdoH
E. coli, mutant mdoH
E.
log2MIC of S. enterica mdoH mutants in Rifampicin
p>0.999
log2MIC
S. enterica wild-type mdoH
S. enterica mutant mdoH
F.
Growth rates of S. enterica mdoH mutants in Rifampicin
p=0.4795
p=0.4795
Monoculture growth rates
Co-culture growth rates
S. enterica, wild-type mdoH
S. enterica, mutant mdoH
S. enterica, wild-type mdoH
S. enterica, mutant mdoH

S3 fig. Impact of *mdoG* and *mdoH* mutations on MICs and growth rates in rifampicin-resistant evolved *E. coli* and *S. enterica*. Each data point represents the average MIC for three isolates obtained from a single population. For each species- culture type combination, there are six populations total, and the statistical comparisons represent MIC comparisons between populations with wild type vs. mutant alleles. **A.** MIC of *mdoG* wild-type vs. mutant *E. coli* isolates. **B.** Monoculture and co-culture growth rates of *mdoG* wild-type vs. mutant *E. coli* isolates. **C.** MIC of *mdoH* wild-type vs. mutant *E. coli* isolates. **D.** Monoculture and co-culture growth rates of *mdoH* wild-type vs. mutant *E. coli* isolates. E**.** MIC of *mdoH* wild-type vs. mutant *S. enterica* isolates. **F.** Monoculture and co-culture growth rates of *mdoH* wild-type vs. mutant *S. enterica* isolates.
