# Supplementary material for: Cross-feeding modulates the rate and mechanism of antibiotic resistance evolution in a model microbial community of Escherichia coli and Salmonella enterica
Source: PLoS Pathog. 2020 Jul 20;16(7):e1008700. doi: 10.1371/journal.ppat.1008700 (PMC7392344; doi:10.1371/journal.ppat.1008700)
Supplement: S4 Fig — Each data point represents the average MIC for three isolates obtained from a single population. For each species- culture type combination, there are six populations total, and the statistical comparisons represent MIC comparisons between populations with wild type vs. mutant alleles. A. MICs of wild-type vs. mutant E. coli with mutations in rne (monoculture-evolved, in blue) and proQ (coculture-evolved, in green). B. MICs of wild-type vs. mutant S. enterica with mutations in acrB/ompR, ramR, (all evolved in monoculture only), and metL (evolved in co-culture only). C. MICs of wild-type vs. mutant S. enterica with mutations in ompF evolved in monoculture (gold) or co-culture (green). D. MICs of wild-type vs. mutant S. enterica with mutations in yoaE evolved in monoculture (gold) or co-culture (green). (PDF) [file ppat.1008700.s006.pdf]

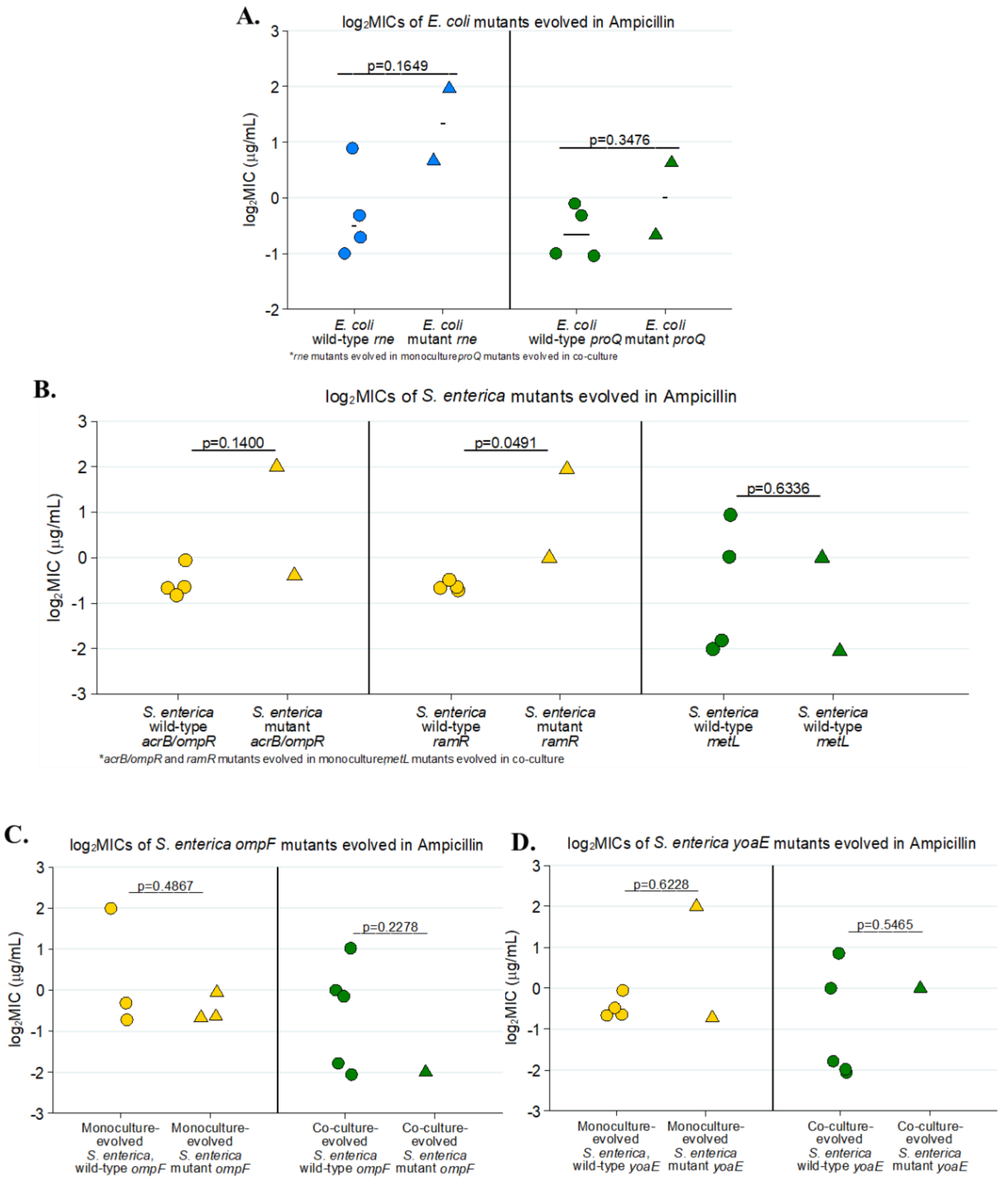

S4 fig. Effect of other mutations on MICs of ampicillin-evolved isolates. Each data point represents the average MIC for three isolates obtained from a single population. For each species- culture type combination, there are six populations total, and the statistical comparisons represent MIC comparisons between populations with wild type vs. mutant alleles. **A.** MICs of wild-type vs. mutant *E. coli* with mutations in *rne* (monoculture-evolved, in blue) and *proQ*

(coculture-evolved, in green). **B.** MICs of wild-type vs. mutant *S. enterica* with mutations in *acrB/ompR*, *ramR,* (all evolved in monoculture only), and *metL* (evolved in co-culture only). **C.** MICs of wild-type vs. mutant *S. enterica* with mutations in *ompF* evolved in monoculture (gold) or co-culture (green). **D.** MICs of wild-type vs. mutant *S. enterica* with mutations in *yoaE* evolved in monoculture (gold) or co-culture (green).
