# Supplementary material for: Cross-feeding modulates the rate and mechanism of antibiotic resistance evolution in a model microbial community of Escherichia coli and Salmonella enterica
Source: PLoS Pathog. 2020 Jul 20;16(7):e1008700. doi: 10.1371/journal.ppat.1008700 (PMC7392344; doi:10.1371/journal.ppat.1008700)
Supplement: S5 Fig — P = 0.0614 for monocultures, p = 0.3545 for co-cultures, Mann-Whitney U test. Each data point represents the average MIC for three isolates obtained from a single population. For each culture type combination, there are six populations total, and the statistical comparisons represent MIC comparisons between populations with wild type vs. mutant alleles. (PDF) [file ppat.1008700.s007.pdf]

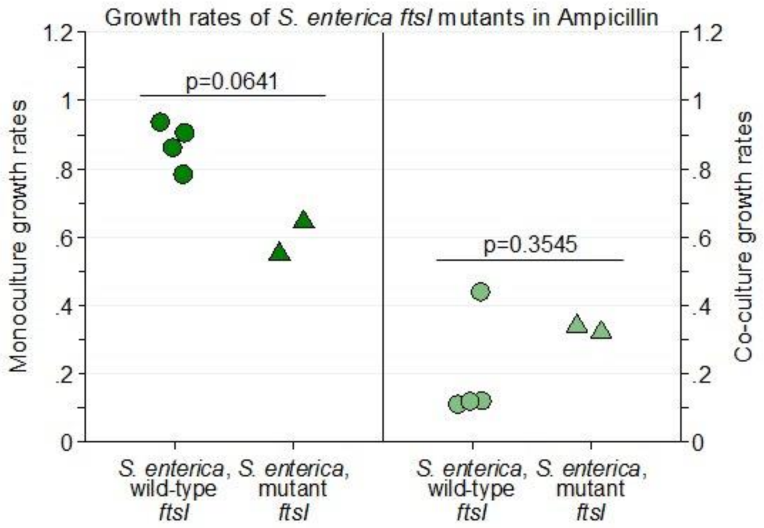

S5 fig. Monoculture and co-culture growth rates of *ftsI* mutant isolates in pH=4.7 growth medium. P= 0.0614 for monocultures, p= 0.3545 for co-cultures, Mann-Whitney U test. Each data point represents the average MIC for three isolates obtained from a single population. For each culture type combination, there are six populations total, and the statistical comparisons represent MIC comparisons between populations with wild type vs. mutant alleles.
