# Supplementary material for: Cross-feeding modulates the rate and mechanism of antibiotic resistance evolution in a model microbial community of Escherichia coli and Salmonella enterica
Source: PLoS Pathog. 2020 Jul 20;16(7):e1008700. doi: 10.1371/journal.ppat.1008700 (PMC7392344; doi:10.1371/journal.ppat.1008700)
Supplement: S6 Fig — Error bars represent standard deviation. (PDF) [file ppat.1008700.s008.pdf]

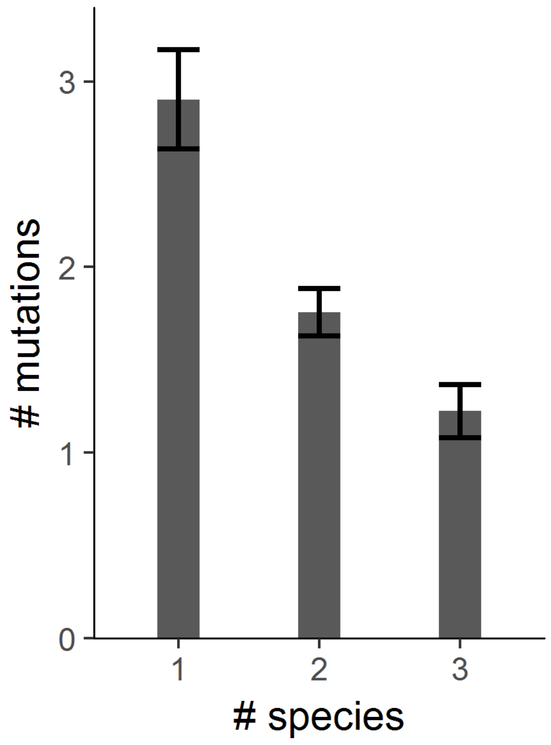

S6 fig. Average number of mutations that were observed in simulations with increasing numbers of species. Error bars represent standard deviation.
