# Supplementary material for: Cross-feeding modulates the rate and mechanism of antibiotic resistance evolution in a model microbial community of Escherichia coli and Salmonella enterica
Source: PLoS Pathog. 2020 Jul 20;16(7):e1008700. doi: 10.1371/journal.ppat.1008700 (PMC7392344; doi:10.1371/journal.ppat.1008700)
Supplement: S7 Fig — Average MIC of isolates from each evolved rifampicin (A) and ampicillin (B) population. Numbers next to the data points represent population numbers. High-frequency (>50%) genotypes can be found in S1 Table. P-values represent Mann-Whitney U test results. (PDF) [file ppat.1008700.s009.pdf]

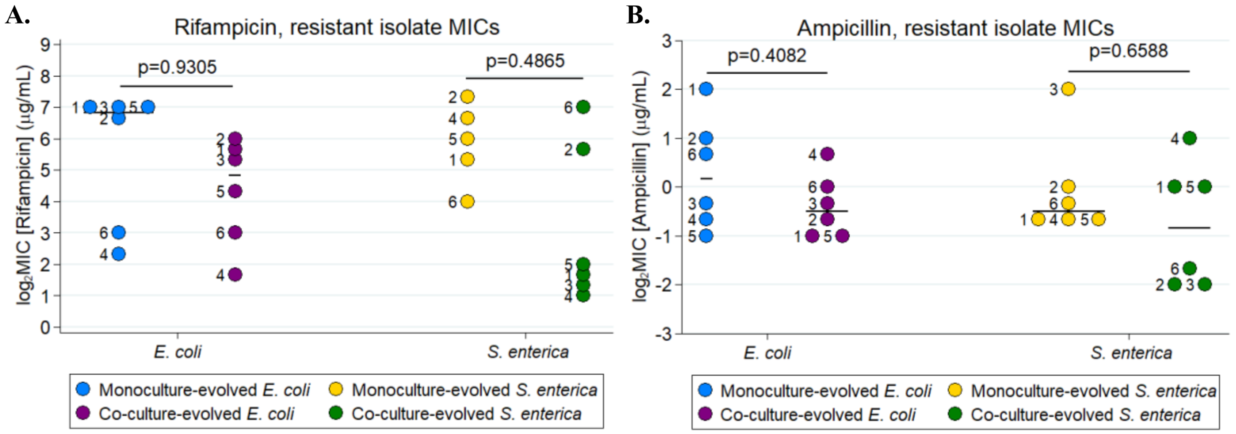

S7 fig. Average MIC of isolates from each evolved rifampicin (**A**) and ampicillin (**B**) population. Numbers next to the data points represent population numbers. High-frequency (>50%) genotypes can be found in **S1 table**. P-values represent Mann-Whitney U test results.
